# The Evolution of Diapsid Reproductive Strategy with Inferences about Extinct Taxa

**DOI:** 10.1371/journal.pone.0158496

**Published:** 2016-07-08

**Authors:** Jason R. Moore, David J. Varricchio

**Affiliations:** 1 Honors College, University of New Mexico, Albuquerque, New Mexico, United States of America; 2 Department of Earth Sciences, Montana State University, Bozeman, Montana, United States of America; New York Institute of Technology, UNITED STATES

## Abstract

Diapsids show an extremely wide range of reproductive strategies. Offspring may receive no parental care, care from only one sex, care from both parents, or care under more complex regimes. Young may vary from independent, super-precocial hatchlings to altricial neonates needing much care before leaving the nest. Parents can invest heavily in a few young, or less so in a larger number. Here we examine the evolution of these traits across a composite phylogeny spanning the extant diapsids and including the limited number of extinct taxa for which reproductive strategies can be well constrained. Generalized estimating equation(GEE)-based phylogenetic comparative methods demonstrate the influences of body mass, parental care strategy and hatchling maturity on clutch volume across the diapsids. The influence of polygamous reproduction is not important despite a large sample size. Applying the results of these models to the dinosaurs supports the hypothesis of paternal care (male only) in derived non-avian theropods, previously suggested based on simpler analyses. These data also suggest that sauropodomorphs did not care for their young. The evolution of parental-care occurs in an almost linear series of transitions. Paternal care rarely gives rise to other care strategies. Where hatchling condition changes, diapsids show an almost unidirectional tendency of evolution towards increased altriciality. Transitions to social monogamy from the ancestral state in diapsids, where both sexes are polygamous, are common. In contrast, once evolved, polygyny and polyandry are very evolutionarily stable. Polygyny and maternal care correlate, as do polyandry and paternal care. Ancestral-character estimation (ACE) of these care strategies with the character transition likelihoods estimated from the original data gives good confidence at most important nodes. These analyses suggest that the basalmost diapsids had no parental care. Crocodilians independently evolved maternal care, paternal care evolved in the saurischian line, prior to derived theropod dinosaurs, and the most basal neognaths likely exhibited biparental care. Overall, the evolution of parental care among diapsids shows a persistent trend towards increased care of offspring, and more complex care strategies and behaviors with time. Reversions to reduced care are infrequent.

## Introduction

Extant diapsids (a diverse group of tetrapods including snakes, lizards, turtles, crocodiles, birds, and their extinct relatives, such as dinosaurs, pterosaurs, ichthyosaurs, etc.) exhibit a wide range of reproductive strategies. Diapsids show marked variations in the level of development of young at hatching, the number of young in a clutch, the sex(es) caring for the offspring before and after hatching, and the contribution of more than one individual of either sex to the clutch, to name but a few. The phylogenetically widespread, complex distribution of many derived reproductive strategies suggests that such strategies evolved early in the diapsids, but that the benefits of each strategy are situational. Evidence of taxa with derived reproductive ecologies from the fossil record is uncommon (e.g. [1–4]).

The evolution of parental care strategies in non-avian diapsids is relatively little studied [5–7], potentially because these taxa, with the exception of crocodilians, are dominated by the absence of parental care and superprecocial young–the primitive states for diapsids [5,8]. In lizards and snakes, pre-hatching care (either nest attendance, nest guarding, or, occasionally, the brooding of eggs) is somewhat rare (~2% of species), whereas post-hatching care of any kind is extremely uncommon, if not absent [5]. The 20 extant species of crocodilians, however, almost uniformly show some form of pre-hatching care, and the majority also show either transport or defense of hatched young [5]. Nevertheless, quantitative analyses of the evolution of care strategies in these taxa are few, and many of these studies have not utilized some of the more modern advances in evolutionary analysis [8].

Reproduction in extant birds differs yet farther from non-avian diapsids. Important differences include: hard calcitic eggshell with multiple structural layers; large, relative to adult body size, and commonly asymmetric eggs; sequential ovulation leading to iterative egg laying; nearly universal parental care of eggs and young; and adult-egg contact incubation [9–10]. Ornithologists have long sought to document and explain the evolution of this modern avian reproductive mode. Phylogenetic analysis indicates that several features such as self-feeding, precocial young [11–12], and open nests [13] are ancestral for extant birds. These analyses have not, however, been able to definitively recognize the ancestral mode of parental care for extant birds, with both biparental [6,11,14] and paternal [15] being favored depending on the permitted character transitions and the use or lack of an outgroup. Other arguments and lines of evidence support maternal [16–19], biparental [20], or paternal [21–25] care as the ancestral state. The ambiguity of the ancestral state in part stems from the prevalence (but not necessarily the ancestral condition) of biparental and paternal care in the two major clades of extant birds, Neognathes and Palaeognathes, respectively.

Non-avian dinosaurs occupy a key phylogenetic position, between birds and crocodiles, and may help resolve the ambiguities of the state of basal avian reproductive ecologies, particularly by providing data regarding the evolution of those care strategies (i.e. paternal care) that are novel in birds. Evidence of derived reproductive strategies has been found from two major clades of dinosaurs: the duck-billed hadrosaurs [1], and the predominantly carnivorous theropods [26–30].

Based on cohorts of neonates of significantly varying sizes associated with different hatched nests, hadrosaurs, specifically the species *Maiasaura peeblesorum*, have been suggested to have cared for their subaltricial young at the nesting site for an extended period after hatching [1,31–32]. Such behavior would likely be linked to both pre-hatching nest attendance and presumably more complex post-hatching care.

Oviraptorids, troodontids, and dromaeosaurids are the dinosaur clades sharing the closest ancestry with birds [33]. For the first two clades, a surprisingly wide array of reproduction-related fossils exists, including eggs, embryos, clutches, nests, and clutch-associated adults. These specimens demonstrate that reproduction in these theropod dinosaurs differed widely from that of other non-avian dinosaurs, possessing many attributes common to modern birds, e.g. eggshell microstructure, egg shape, and sequential ovulation, and that these traits trace their origins to these Mesozoic dinosaurs [27,30,34–38].

More controversy surrounds the interpretation of the behavioral and evolutionary significance of oviraptorid and troodontid adults found atop egg clutches. Some [26,28–29,39–40] argue that based on posture, the likely presence of a feather integument, the temperature requirements of synchronous hatching given iterative laying, and the avian-like levels of egg porosity, that these dinosaurs engaged in brooding with or without contact incubation as in birds. Others argue that these specimens represent more reptilian and crocodile-like nest-guarding with soil incubation given the limited contact of adult and eggs, the lack of egg rotation, and the significant portion of eggs remaining buried in sediments within the nest [9,41–44]. This controversy can be stated as a question of homology. Do these specimens provide evidence of parental care in these theropods that is homologous to that of modern crocodilians, birds, both or neither? Implicit within this question is whether the parental care of these non-avian dinosaurs represents biparental, maternal, or paternal care. Answering these questions has implications for both the evolution of the modern avian reproductive mode and reproduction in other dinosaur clades of the Mesozoic.

For their adult body mass, both oviraptorids and troodontids possess anomalously large clutches in comparison to those of extant reptiles, most extant birds, and other non-avian dinosaurs [45]. Horner [46] suggested that troodontid clutches might reflect communal nesting and Wesołowski [24], in modeling the evolution of modern avian reproduction, proposed a communal nesting stage with paternal care. Further, Vehrencamp [15] demonstrated that among extant birds, male incubation represents a necessary precursor for communal laying. In an earlier analysis focused on examining some of the questions raised here, Varricchio et al. [47] undertook a study to test which of four care models the unusually large clutches of these dinosaurs fit best. Among the alternatives of crocodilian maternal and avian biparental, maternal, or paternal care, the relative clutch size in these oviraptorid and troodontid dinosaurs matched best with the avian paternal care model [47]; potential additional support for this claim was offered by the lack of female-only histological markers in nest-associated adults of these dinosaur species. This conclusion has been contested by some more recent studies [14,48–49].

More recently, Birchard et al. [14] conducted a similar study using a large avian data set that included a large number of Anseriforms, but also excluded megapodes, one of the few extant clades with some paternal care of eggs [50]. They found that among extant birds, relative clutch mass scaled similarly among those taxa with paternal and maternal care and differed from those with biparental care. Birchard et al. [14] also note that in the Charadriiformes, their only higher taxon showing maternal, paternal and biparental care, the taxa showing paternal care have smaller clutches than taxa with other care strategies. The non-avian dinosaur data of Varricchio et al. [47] plotted with the maternal and paternal regressions in this more recent study. Birchard et al. [14] concluded, therefore, that the "type of parental care cannot be distinguished by conventional allometry because of the confounding effects of phylogeny and hatching maturity" [14]. Consequently, they favored biparental care as the ancestral condition for extant birds based on the phylogenetic distribution of care. But this phylogeny included no outgroups and disregarded their own findings that the included non-avian dinosaurs possessed either paternal or maternal but not biparental care. Birchard et al. [14] also suggested that some of the variation in clutch volume in extant birds is associated with clade-specific ecological factors that are unaccounted for by these general analyses. This last conclusion was supported by Watson et al. [51], who demonstrated that megapodes have a significantly larger clutch volume than expected, as they use environmental heat, rather than body heat, to incubate their eggs.

A further test for hypotheses of uniparental care among diapsids is offered by the observation by Bennett and Owens [52] that social polygamy (i.e., both polygyny and polyandry) in birds is commonly associated with uniparental care by the non-deserting parent; the reproductive costs of one parent leaving are less than the benefit gained by that parent from additional mating opportunities, or simply from relief from the burden of care. They posit that breeding density influences nest desertion: Low density nesting favors female desertion, as males are unlikely to find another fertile female; high density nesting favors male desertion, as they can desert immediately on fertilization, leaving the females to care for the clutch out of necessity. According to this hypothesis, polygyny/polyandry is closely tied to parental care strategy and so maternal and paternal care should correlate with polygyny and polyandry, respectively.

Here we broaden the approach of Varricchio et al. [47] to address the question of the evolution of reproductive strategies across the diapsids, with a more refined analysis that incorporates both of the "confounding effects of phylogeny and hatchling maturity" of Birchard et al. [14]. Major changes relative to our initial analysis [47] include: 1) a new regression analysis using the methods detailed by Paradis [53] and incorporating a fully resolved phylogeny down to the species level; 2) inclusion of a number of non-archosaurian taxa, to provide a broader perspective on diapsid reproductive ecology in general, and to better resolve the reproductive ecologies of the dinosaurs; and 3) an expanded dataset including all non-avian dinosaurs with suitable data. This is still a very limited sample of non-avian dinosaurs, but their inclusion begins to address the homology of parental care found in crocodilians, maniraptoran theropods, and birds. Finally, 4) we modified our criteria for the coding of parental care to more accurately reflect the potential contributions of both sexes. In Varricchio et al. [47] and Birchard et al. [14], parental systems reflect attending, guarding and or incubating of eggs, nests or incubation structures. The new criterion underscores alternative contributions of mates to reproduction during the incubation period. For example, the emperor penguin, *Aptenodytes forsteri*, maintains a monogamous pair bond through the incubation of their single egg. Although the male incubates the egg, the female will eventually return to provision and relieve the male [54]. Consequently, the male's effort is dependent upon that of the female. We feel such cases are more accurately scored as biparental.

## Materials and Methods

No permits were required for the described study, which complied with all relevant regulations. All fossil data were collected from specimens already curated in publically accessible permanent repositories. Locality information is available to qualified researchers from the specimens’ repositories. All analyses were carried out in R 3.1.3 [55] using the *ape* and *nlme* packages.

### Reproductive Strategy Data

To test the hypotheses described above, we gathered data on the following variables for the included species: adult body mass, clutch volume, hatchling precociality, mating strategies, parental care mode, and higher taxonomic group, categorized either by order, or by whether the taxon was a megapode (Table 1, S1 File). Categories of hatchling precociality, i.e. altricial through superprecocial, followed the definitions in Gill [56]. Mating strategies represent *social* mating systems [57].

**Table 1:** Coding of reproductive ecological traits used in these analyses.

Reproductive Strategy Trait	Value/State
Body Mass (BM)	kg
Hatchling Precociality (HP)	Superprecocial (coded as 0), Precocial (coded as 1), Semiprecocial (coded as 2), Subaltricial (coded as 3), Altricial (coded as 4)
Polygyny (FP)	Present/Absent [52]
Polyandry (MP)	Present/Absent [52]
Parental Care Strategy (CS)	No Care, Paternal Care, Maternal Care, Biparental Care
Higher Taxon (HT)	~Order (see S1 File for details)
Megapodes (ME)	Yes/No (see S1 File for details)

In response to criticism of the classification of the ostrich, *Struthio camelus*, as showing paternal care in prior analyses [14,47] we note that a polygynous male initially attends the clutch from multiple females alone prior to incubation [57], and so it could be coded as either paternal or biparental, depending on the proportion and type of care provided by the females necessary for care to be considered biparental. All analyses presented here were undertaken twice, once with *S*. *camelus* coded as showing paternal care, and once as biparental care. The coding of parental care strategy in *S*. *camelus* made no difference to the outcomes of any of our analyses, so, in the interests of clarity, we will only present the results of the analyses with *S*. *camelus* coded as biparental.

#### Extant Taxa

The total data set represents 472 extant diapsids, including 37 lepidosaurs, 11 chelonians, 15 crocodilians with maternal care and 4 crocodilians with biparental care, 330 birds with biparental care, 30 birds with maternal care and 44 birds with paternal care. A variety of sources provided the reproductive and body mass data for the analyses. Egg dimensions, reproductive behaviors, hatchling condition, etc. for modern birds primarily came from Walters [58] and The Handbook of the Birds of the World [59]. Additional sources provided information on megapodes [50], ratites and tinamous [57], *Actitis macularius* [60], and *Monias benschi* [61]. For adult avian weight we used the species average or the average of both sexes as reported in the above references or in Dunning [62] where available. Clutch volume was calculated using the equation for avian egg volume, V = 0.51 L x D^2^ where L = egg length and D = egg diameter [63], multiplied by the average number of eggs per clutch. In a few instances, e.g. *Jacana jacana*, *Eudromias morinellus*, and *Phalaropus lobatus*, clutch mass data [64] was converted to clutch volume using a standard bird egg density of 1.06 g/cm^3^ [65], which matches an average of egg density for 28 extant bird species [66].

Ferguson [67] provides egg dimensions for extant crocodilians. Egg volume was calculated using the equation for an ellipse (V = 0.524 L x D^2^). Data on crocodilian eggs per clutch and adult female weights came from Thorbjarnarson [68]. Trutnau and Sommerlad [69] provided data on *Caiman yacare* and *Osteolaemus tetraspis*. For three crocodilians, *Paleosuchus trigonatus*, *Crocodylus intermedius*, and *Crocodylus moreletti*, clutch mass was converted to volume using an average crocodilian egg density of 1.13 g/cm^3^ ([67] table III). Information on crocodilian parental care came from Shine [5] and Trutnau and Sommerlad [69]. Most crocodilians exhibit female nest attendance during incubation, but several taxa, e.g. *Caiman crocodilus*, *Crocodylus niloticus*, and *C*. *palustris* among others, exhibit biparental care of young.

Somma [70] and Köhler [71] provided information on reproductive behaviors and data for both lepidosaurs and chelonians. Additional data was collected on individual species from a collection of journal articles (S2 File).

#### Fossil Taxa

Reproductive and body mass data were collected for the nine dinosaur taxa for which reliably identified, well-described clutches have been described. These comprise four maniraptoran theropods (*Troodon formosus*, *Byronosaurus jaffei*, *Machairasaurus philoceratops*, *and Citipati osmolskae*), and five additional dinosaur taxa: the early Jurassic prosauropod *Massospondylus* [72–73]; two Late Cretaceous ootaxa, *Megaloolithus patagonicus* and *Megaloolithus sirugei*, assigned to titanosaur sauropods on the basis of embryos in the former [74–75]; and two hadrosaurs, an unknown lambeosaurine and *Maiasaura peeblesorum* [76–77]. Note that the Jurassic allosauroid theropod, possibly *Lourinhanosaurus*, with well described eggs from Portugal [78–79] was excluded due to significant ambiguity in clutch size among published accounts.

All examined *Troodon formosus* material comes from the Upper Cretaceous (Campanian) Two Medicine Formation of western Montana and are curated at the Museum of the Rockies (MOR), Bozeman, Montana. *Troodon* egg dimensions and volume were based upon Varricchio et al. [28]. MOR 363 and 963 represent the most complete egg clutches for *Troodon* and contain 22 and 24 eggs respectively [1,27,80–81]. Adult size for *Troodon* was calculated using the minimum femoral circumference from MOR 553S-7-16-0-61 and an allometric equation [82]. MOR 553S-7-16-0-61 represents the largest known *Troodon* femur and predicts a weight of 51.4 kg. This exceeds all previous *Troodon* weight estimates of 45.3 kg [83], 41.9 kg [84], 50 kg [85], and 40 kg [86]. In comparison, the circumference of MOR 748, a clutch-associated adult [27], translates to a body mass of only 33.6 kg. Consequently, the clutch volume to adult mass ratio likely represents a conservative estimate.

Egg and clutch data for *Byronosaurus jaffei* were based on observation of Mongolian Institute of Geology, Ulaan Baatar (IGM) 100/1003. A similar sized deinonychosaurian, *Saurornitholestes* [87] provides an approximate adult body mass.

Egg size (55 x 150 mm) and femur dimensions (diameter = 30 mm) for *Machairasaurus philoceratops* come from the description of IVPP V9608 (Institute of Vertebrate Paleontology and Paleoanthropology, Beijing, China), a clutch-associated adult from the Upper Cretaceous Djadokhta Formation of Inner Mongolia, China [88]. The predicted adult weight of 39 kg based on the allometric equation for femur circumference [82] compares favorably to other mass estimates of 33 kg [85] and 40 kg [86]. Complete *Machairasaurus* clutches are estimated to contain 30 or more eggs [88–90].

Good embryonic specimens exist for multiple oviraptorid and troodontid taxa that indicate through both overall skeletal development and histologic examination that both clades possessed precocial young [32,91–96].

Norell et al. [26] and Clark et al. [29] provide information for *Citipati osmolskae* including egg dimensions and clutch size. For *Citipati osmolskae*, we used the egg dimensions of 65 x 180 mm [26], which are comparable to others reported [29–30]. Because the eggs are largely symmetrical, egg volumes for both *Machairasaurus* and *Citipati* were calculated using the equation for an ellipse. Although only 15 eggs were visible in association with IGM 100/979, estimates of clutch size for this specimen range from 22 to 30 eggs [26,29]. We used the lower estimate to calculate clutch volume. Femoral mid-shaft circumference was not available for IGM 100/979. Adult body mass was instead estimated from a femoral circumference of 121.9 mm as measured on IGM 100/1004, a second and larger clutch-associated *Citipati* [97].

Adult body mass in *Massospondylus* was calculated using the methods of Henderson [98]. Body masses for the adults associated with *M*. *patagonicus* and *M*. *sirugei* were estimated as 5000 and 10000 kg respectively, from Sander [75]. Note that we use data from specimens referred to *M*. *sirguei* only, but that this taxon has been synonymized with *M*. *mamillare* across Spain and southern France [75]. *M*. *sirguei* represents a subset of variation within *M*. *mamillare*, and so we opt to keep the former designation in this study to facilitate accurate comparison of our data. All show a relatively smaller clutch volume to adult mass ratio than the troodontids and oviraptorids.

Clutch volumes for the lambeosaurine (assigned to *Hypacrosaurus sp*.) and *Maiasaura* clutches were calculated from Horner [1,77]. Body mass estimates for *Hypacrosaurus* and *Maiasaura* were calculated from Anderson et al. [82] and Dunham et al. [99], respectively.

There is insufficient data on the presence or absence of polygamy in any of the fossil taxa used here to allow for the incorporation of these in analyses of the evolution of polygamy, so all such conclusions are be based on extant data only. Similarly, all fossil taxa are assumed to have precocial offspring, except the hadrosaurs, some of which have been suggested to be subaltricial [1,31].

### Phylogeny

Several hypotheses for the relationships of the major diapsid groups have been suggested recently, differing only in the placement of the chelonians among the other diapsid clades: the traditional view, originally based on morphological analyses, but now with some molecular support, places chelonians as the sister group to diapsids, with squamates as the sister group to archosaurs [100–101]; whereas more recent hypotheses based primarily on molecular analyses, place chelonians either as the sister group to lepidosaurs (e.g. [102]) or suggest that squamates are the most basal diapsid group, with chelonians as the sister group to archosaurs [103]. While there appears to be increasing support for the latter hypothesis, for the sake of completeness we repeated the analyses contained herein for all tree topologies. Choice of tree topology has no effect on the interpretation of the results of these analyses, so only the results of analyses using the “squamates basal-chelonians derived” phylogeny [103] are presented here.

The phylogeny used for all our analyses was built upon the recent avian tree of Jarvis et al. [104] and secondarily from that of Hackett et al. [105]. But as these trees resulted from analyses using only 48 and 169 species, respectively, they only provided a topology for the major clades. As methods of this study (see below) require a fully resolved phylogeny at the species level, we drew upon a diverse array of more phylogenetically focused analyses to place all the 472 species used in this study (S3 File). Because these subordinate trees, e.g. the cranes, crakes and rails (Gruiformes) [106], could typically be placed with the phylogenies of Jarvis et al. [104] or Hackett et al. [105], we did not undertake a supertree analysis. The complete phylogeny is shown in S4 File.

### Assessing the influence of reproductive strategy on clutch volume

Regression techniques have been used to correlate changes in the mass or volume of a laid clutch with different reproductive strategies of the adults contributing to the clutch, whilst accounting for the influence of body mass [47,52,107]. To date, however, these approaches have not simultaneously addressed the non-independence of traits associated with phylogenetic proximity (i.e. taxa that are more closely related are more likely to show a similar/identical character trait than those only distantly related–an issue that significantly reduces the strength of traditional regression techniques for this style of analysis due to violation of the assumption of independence between observations) and the complexities of analyzing ordinal or categorical data (i.e. mating systems being polygamous or not) within a regression framework. To address both of these issues in a single analysis, we use Generalized Estimating Equations (GEEs [108], for details see [48]) to quantify the influence of a range of reproductive traits (both continuous and discrete) on diapsid clutch volume while accounting for phylogenetic non-independence. An additional advantage of the use of GEEs is that phylogenetic correlation structures based on different models of evolution (i.e. Brownian [109], Ornstein-Uhlenbeck [110]) can be incorporated and compared in GEE analyses [48]. Note that while the use of GEE accounts for phylogenetic non-independence, it does not automatically account for any character trait patterns associated with particular clades. Instead individual analyses testing for separate patterns within the assigned higher taxa must be undertaken to assess these patterns.

Several steps of GEE model selection were undertaken to assess which of our defined reproductive traits influenced clutch volume and which model of evolution best describes the transitions between states of these traits. Initially, complete models were developed relating all of our measured traits (body mass, level of hatchling precociality, presence of male or female polygamy in a species, and parental care strategy) to clutch volume under Brownian and Ornstein-Uhlenbeck evolutionary models. The complete data used for these analyses can be found in S1 File. Based on existing theoretical predictions [47,52], the following subsets of factors from the complete model have been hypothesized to influence clutch volume: body mass only; body mass and care strategy only; body mass and care strategy plus precociality; and body mass and care strategy plus polygamy. To test those hypotheses, we ran each developed a GEE model for each subset or potentially important variables. Finally, to test for any clade-specific clutch volume patterns (caused by unaccounted-for ecological variation, e.g. [14]) we reran the complete model and each of the four subset models adding higher taxon as an additional factor (after Birchard et al. [14]), and also adding a factor differentiating megapodes from the remaining taxa. Megapodes have been specifically suggested to have a significantly larger clutch volume than other similar taxa [51]. We have only two megapodes (*Leipoa ocellata* and *Alectura lathami*) in our dataset, and they do show markedly larger clutches. As these are some of the few taxa that show paternal care, it is important to ensure this outlier does not affect our overall diapsid care strategy model. These additions give a total of 15 models to be compared (Table 2).

**Table 2:** Summary of variables included in GEE models used to predict clutch volume analyses.

i) BM	vi) BM + HT	xi) BM + ME
ii) BM + CS	vii) BM + CS + HT	xii) BM + CS + ME
iii) BM + HP + CS	viii) BM + HP + CS + HT	xiii) BM + HP + CS + ME
iv) BM+ MP + FP + CS	ix) BM+ MP + FP + CS + HT	xiv) BM+ MP + FP + CS + ME
v) BM + HP + FP + MP + CS	x) BM + HP + FP + MP + CS + HT	xv) BM + HP + FP + MP + CS + ME

Acronyms refer to included reproductive ecological traits from Table 1.

To test the observations of Birchard et al. [14] that, unlike the pattern predicted for most birds, Charadriiformes with paternal care had smaller clutches than Charadriiformes with other care strategies, we repeated the GEE analyses above on a subset consisting of the 66 Charadriiformes. There are insufficient Charadriiform taxa showing maternal care to allow analysis of that care strategy, but subset GEE analysis examining the influence of biparental and paternal care is possible.

All calculated models were compared using quasi-likelihood information criterion (QIC [111]), to determine which produced the best fit to the data with the least variables. The model with the lowest QIC value produces the best balance of model complexity to explanatory power, and all models within 2 QIC of the lowest QIC model have similarly strong levels of explanatory power. Models within 4 QIC have moderate explanatory power. If multiple models have similarly strong explanatory power, and parameter estimates are required, model averaging can be used to produce a more robust estimate of the model parameters [112]. Model averaging is carried out by summing the parameter estimates for each variable for each model in which they are included, weighted by the QIC weights (equivalent to the Akaike weights described below) of the model [112]. We use the second method of Burnham and Anderson [112] to carry out model averaging, considering each variable included in all models, but with its parameter estimate set to zero in model subsets without that variable. QIC [113] is an extension of the more frequently used and more widely applicable Akaike Information Criterion [114], which cannot be used within a GEE framework, as GEE is quasi-likelihood based rather than maximum likelihood based [111].

Once the most likely GEE model has been established, it is possible to substitute in known values of all of the included reproductive ecological traits from extinct diapsids to that model, and use them to make a prediction of clutch volume under each potential parental care strategy (in a similar manner to Varricchio et al. [47]). The variance from the expected clutch volume can be used to calculate the Akaike Information Criterion (AIC [114]) for each model [112]. As with QIC, AIC is an estimate of the information lost when the model is used to approximate reality, and the lowest AIC value represents the best model amongst a tested set, providing that all models have been generated from exactly the same dataset [112]. From the differences in AIC values (Δ_i_), it is possible to calculate the Akaike Weights (*w*_*i*_) of each model (*w*_*i*_ = *e*^-0.5Δi^/*∑e*^-0.5Δi^), and hence the evidence ratios (*w*_*i*_*/w*_*j*_, where *w*_*j*_ is the most likely model) showing how many times less likely each alternative model is than the best [112].

### Estimating ancestral reproductive strategies

It is also possible to use the phylogenetic distribution of reproductive strategies (i.e. the characters listed in Table 1) amongst terminal taxa to estimate the likelihood that a given node within the phylogeny holds a particular character state; i.e. estimate the character state of the last common ancestor between any pair of taxa [115]. This allows for the investigation of the evolutionary history of reproductive strategies, in comparison to the opportunity to study the evolutionary mechanics of these strategies provided by the GEE analyses detailed earlier. Two ancestral character estimation (ACE) analyses are possible using the data gathered/generated so far. The first analysis considers only the phylogeny of extant diapsids used as the basis for the analyses in the previous section. The second analysis incorporates the parental care strategies predicted for the extinct taxa by the GEE models detailed earlier, as well as any other extinct taxa for which good data exist. We recognize that the latter approach introduces an added level of uncertainty into the interpretation of the ACE analyses, but the large phylogenetic gaps created by using the extant only dataset could lead to an oversimplified view of character evolution (as highlighted by Betancur-R et al. [116]). Consequently, we present and contrast both analyses.

The first step in such an ACE analysis is to generate a theoretical character transition model [53]. Such a model defines which character states can transition to which others (i.e. can state A transition to either states B or C, or must transitions proceed A to B to C), and whether these transitions occur with similar or different likelihoods (i.e. is the likelihood of transitioning from A to B the same or different as that from B to C). It is possible to build a very large number of potential character transition models, but best practice suggests that only those with strong logical or theoretical support should be tested.

With the character transition models defined, and the distribution of character states established amongst taxa in a chosen phylogeny and branch lengths assigned (all branch lengths were set to 1 in these analyses, as branch lengths were incomparable between the phylogenies compiled to produce the whole diapsid tree), it is then possible to estimate the likelihoods of each character state transition, and so the character states at each node within the phylogeny using a continuous-time Markov model [117]. As the reproductive data collected here include discrete characters, the only suitable method to estimate ancestral character states is maximum likelihood [117], rather than least squares, residual maximum likelihood or generalized least squares [53], all of which are only applicable to continuous data. The relative quality of each candidate transition model can be assessed using AIC, as detailed above.

Three character transition models were generated to test a range of hypotheses regarding the evolutionary transitions associated with parental care strategy (Table 3): a uniform model where all characters could transition to all other characters and all transition rates were the same; a symmetrical model where all characters could transition to all other characters but the rates between different pairs of characters could vary; and a custom model where only a subset of transitions was allowed (Biparental<->Maternal, Biparental<->Paternal, Maternal<->No Care, and Paternal<->No Care) and the rates between different pairs of characters could vary (after Tullberg et al. [6]). While many other character transition models are possible, as this is an early attempt at quantifying the likelihoods of these character transitions, we chose to include only these three models as there is limited *a priori* justification for the inclusion of other types of model. Asymmetrical models (i.e. where Biparental->Maternal has a different transition rate to Maternal->Biparental), for example, were not considered, as there is not unambiguous theoretical justification for this approach. The extant-only parental care ACE analysis was undertaken using only the taxa listed in the S1 File. The extinct and extant analysis uses the unambiguously predicted parental care strategies from the previous section to add several extinct dinosaurian taxa to the phylogeny. Moreover, there is some evidence for the absence of parental care in pterosaurs [4], so this group is added. It should be noted, however, that these taxa represent only a fraction of the diversity of dinosaurs, and that the interpretations below are contingent on the accuracy of the predictions of the predictive reproductive model presented in the prior section.

**Table 3:** Tested parental care strategy character transition models.

A) Uniform Model				B) Symmetrical Model				C) Custom Model			
	M	N	P		M	N	P		M	N	P
B	a	a	a	B	a	b	c	B	a	0	b
M		a	a	M		d	e	M		c	0
N			a	N			f	N			d

Lower case letters represent transition rates to be calculated, 0 = no transition possible. Character transitions with the same lower case letter are modeled with the same transition rate. All rates are modeled as equal in A, transition rates are different between different pairs of care strategies in B, and two pairs of transitions (B<->N and P<->M) are disallowed in C. B = biparental care, M = maternal care, N = no care, P = paternal care.

Similarly to the ACE modeling of parental care strategy, three character transition models were built and compared for the five character states coded for hatchling precociality. Only the custom model differed in structure from those described previously, allowing only the following sequential transitions of precociality: Superprecocial<->Precocial<->Semiprecocial<->Subaltricial<->Altricial.

Three possible character transition models were also assessed for fit with the observed diapsid polygamous mating data: a uniform model; a symmetrical model; and a custom model that only permitted a subset of character transitions where only one sex changed polygamy state at a time (i.e., both sexes polygamous<->polygyny, both sexes polygamous<->polyandry, polygyny<->social monogamy, polyandry<->social monogamy).

### Correlation of Care Strategy and Polygamy

According to the hypothesis of Bennett and Owens [52], the presence of polygamy is related to the mode of parental care in birds, hence maternal care should correlate with polygyny, and paternal care with polyandry. We test this hypothesis using one-sided Fisher’s Exact Tests [118] for each instance, assessing the significance of association of polygyny and maternal care, and polyandry and paternal care from 2x2 contingency tables.

## Results

### GEE Model

GEE models were created under Brownian and Ornstein-Uhlenbeck (O-U) modes of evolution, and were subject to the model selection approaches detailed above to determine the most informative subset of parameters modeling clutch volume (Table 4). The O-U mode of evolution consistently fits the data markedly better than the Brownian model.

**Table 4:** Model selection for all tested GEE models under both Brownian and Ornstein-Uhlenbeck modes of evolution using the complete dataset collected here (models from Table 2).

Model	Brownian Evolution QIC	O-U Evolution QIC
i) BM	186.365	***37*.*459***
ii) BM + CS	236.149	***40*.*446***
iii) BM + HP + CS	200.492	***37*.*920***
iv) BM+ MP + FP + CS	239.241	44.073
v) BM + HP + FP + MP + CS	200.102	41.879
vi) BM + HT	453.644	53.225
vii) BM + CS + HT	458.322	58.564
viii) BM + HP + CS + HT	468.529	60.486
ix) BM+ MP + FP + CS + HT	459.464	62.541
x) BM + HP + FP + MP + CS + HT	469.980	64.437
xi) BM + ME	188.630	***37*.*866***
xii) BM + CS + ME	242.280	41.656
xiii) BM + HP + CS + ME	215.036	***39*.*304***
xiv) BM+ MP + FP + CS + ME	245.455	45.204
xv) BM + HP + FP + MP + CS + ME	214.440	43.272

The model with the lowest QIC value (bold) represents the best balance of explanatory power and model complexity available from the suite of tested models. Models within 4 QIC of the best model (bold italics) still contain some explanatory power.

None of the proposed hypotheses was unambiguously supported by the data. Instead, five models (i, ii, iii, xi, and xiii) have some explanatory power (QIC within 4 of the minimum QIC value), and so must be investigated further. Although all five of these could be used in model averaging, we opt not to include models xi and xiii, as these differ only in the state of two data points in a single variable (i.e. that *Leipoa ocellata* and *Alectura lathami* are megapodes). While the low QIC values of these models are suggestive of a unique CV:BM relationship among megapodes, we feel there is insufficient megapode data in these analyses to accurately characterize this relationship, and so these models should be excluded.

Model averaging the remaining three candidate models (i, ii, and iii) was undertaken using the methods of Burnham and Anderson (p.152 [112]). Details of the final, averaged model, and the *β* estimates (regression coefficients quantifying the influence of each factor on clutch volume) for each parameter in the model can be found in Table 5.

**Table 5:** Summary of parameter estimates for the best GEE reproductive strategy model.

Best fit model: Log_10_Clutch Volume = β_1_BM + β_2_PC + β_3_MC + β_4_BC + β_5_HP + I	
Parameter	*β* Estimate
Log_10_ Body Mass (BM)	0.722
Paternal Care (PC)	0.192
Biparental Care (BC)	0.109
Maternal Care (MC)	0.097
Hatchling Precociality (HP)	-0.038
Intercept (I)	5.299

BM in kg; PC, BC and MC coded 0 = absent, 1 = present; HP coded 0–4 (see Table 1).

The *β* estimates indicate that with all other factors held constant, taxa with maternal care will produce the smallest clutches by volume, followed by those with biparental care (although these *β* estimates are within one standard error of each other), with taxa showing paternal care having the largest clutches by a significant margin. All of these are markedly smaller than the clutches produced by taxa without parental care. Similarly, the less precocial (i.e. the more altricial) the offspring produced by a taxon, the smaller the clutch produced.

Repeating the GEE analysis with a subset dataset consisting of only the Charadriiformes (see S1 File) on models i-v (Table 2), shows model i under an O-U evolutionary mode as the most likely model (QIC = 7.29 vs. model ii = 11.345, model iii = 13.221, model iv = 15.338, and model v = 17.218). As model i is the only model with significant explanatory power (i.e. ΔQIC > 4 to the next best model), no model averaging is necessary. Table 6 shows the summary of the parameter estimates of this model. In contrast to the model including all diapsids, Charadriiformes show no influence of parental care strategy on clutch volume.

**Table 6:** Summary of parameter estimates for the best GEE reproductive ecology model fit only to Charadriiformes.

Best fit model: Log_10_Clutch Volume = β_1_BM + I		
Parameter	*β* Estimate	Standard Error
Log_10_ Body Mass (BM)	0.644	0.067
Intercept (I)	5.269	0.056

BM in kg.

Repeating the AIC-based model comparison methods of Varricchio et al. [47] (NB–AIC, rather than QIC is appropriate outside the GEE framework [112]), using an expanded fossil dataset including the recently described troodontid, *Byronosaurus jaffei*, and our new, multivariate, phylogenetically independent model of clutch volume, provides increased support for the conclusions of Varricchio et al. [47]: Paternal care among derived theropods is over three times as likely as the nearest alternative strategy (Table 7, see S5 File for complete data).

**Table 7:** Calculated likelihood that the four theropod dinosaurs *Troodon formosus*, *Byronosaurus jaffei*, *Citipati osmolskae*, and *Machairasaurus philoceratops* show each parental care strategy.

Care Strategy	Sum of Squares	AIC	Δ_i_	Relative likelihood	W_i_	Evidence ratio
No Care	0.491	0.573	4.504	0.105	0.062	9.50
Maternal Care	0.261	-1.321	2.610	0.271	0.161	3.69
Paternal Care	0.110	-3.931	0	1.000	0.592	1.00
Biparental Care	0.238	-1.064	2.327	0.312	0.185	3.20

Two other groups of dinosaurs contain sufficient taxa with complete, identified clutches of eggs to be analyzed in this fashion: the hadrosaurs and sauropodomorphs (Tables 8 and 9, see S5 File for complete data)

**Table 8:** Calculated likelihood that the hadrosaurs for which clutch volume data exist show each parental care strategy.

Hadrosaurs (Lambeosaurine, *Maiasaura peeblesorum*)						
Care Strategy	Sum of Squares	AIC	Δ_i_	Relative likelihood	W_i_	Evidence ratio
No Care	0.371	-0.266	0	1.000	0.293	1.00
Maternal Care	0.407	0.006	0.271	0.873	0.256	1.15
Paternal Care	0.478	0.487	0.753	0.686	0.201	1.46
Biparental Care	0.414	-0.058	0.323	0.851	0.249	1.18

**Table 9:** Calculated likelihood that the sauropodomorphs for which clutch volume data exist show each parental care strategy.

Sauropodomorphs (*Massospondylus carinatus*, *Megaloolithus patagonicus*, *M*. *sirguei*)						
Care Strategy	Sum of Squares	AIC	Δ_i_	Relative likelihood	W_i_	Evidence ratio
No Care	1.210	3.275	0	1.000	0.360	1.00
Maternal Care	1.583	4.083	0.808	0.668	0.241	1.50
Paternal Care	2.003	4.788	1.513	0.469	0.169	2.13
Biparental Care	1.634	4.178	0.902	0.637	0.230	1.57

Neither other group of dinosaurs shows strong evidence for any particular parental care strategy, although sauropodomorphs are at least 50% more likely to show no care than any other care strategy. Consequently, theropods and sauropodomorphs will be included in the remaining extinct-extant analyses, but hadrosaurs will be excluded.

### Ancestral Character Estimation–Care Strategy

Comparing the AIC values for the three possible character transition models for parental care strategy demonstrates that the custom model (i.e. that of Tullberg et al. [6]) is by far the most likely, and so this will be adopted for the remainder of the analyses (Table 10).

**Table 10:** AIC values and evidence ratios for tested parental care strategy character transition models in both extant only dataset and extinct and extant dataset.

	AIC	Evidence Ratio
Extant Only Uniform Model	449.15	1.59 × 10^11^
Extant Only Symmetrical Model	401.56	7.39
Extant Only Custom Model	397.56	1.00
Extant and Extinct Uniform Model	462.01	3.95 × 10^10^
Extant and Extinct Symmetrical Model	417.21	7.39
Extant and Extinct Custom Model	413.21	1.00

Character transition likelihoods can be estimated from the phylogenies for both the extant only and extinct and extant datasets, given the custom transition model (Table 11).

**Table 11:** Character transition likelihoods for custom parental care strategy character transition model.

	B	M	N	P
B		0.0316	0.0000	0.0134
M	0.0303		0.0822	0.0000
N	0.0000	0.0830		0.0000
P	0.0146	0.0000	0.0070	

B = biparental care, M = maternal care, N = no care, P = paternal care. Character transitions in each direction (i.e. B->M and M->B) are the same. Upper right quadrant of the figure gives transitions for the extant-only dataset, lower left quadrant for the extant-extinct dataset.

In general (due to the small number of extinct taxa added) both datasets show similar likelihoods of making each character transition. The transition Maternal<->No Care is the easiest to undertake, followed by Biparental<->Maternal and then Biparental<->Paternal. Maternal<->Paternal and Biparental<->No Care will not occur due to character transition model specification. In the extant-only dataset, the transition Paternal<->No Care also does not occur as determined by the data. When incorporating the extinct data, however, Paternal<->No Care becomes possible, if unlikely.

**Fig 1:**
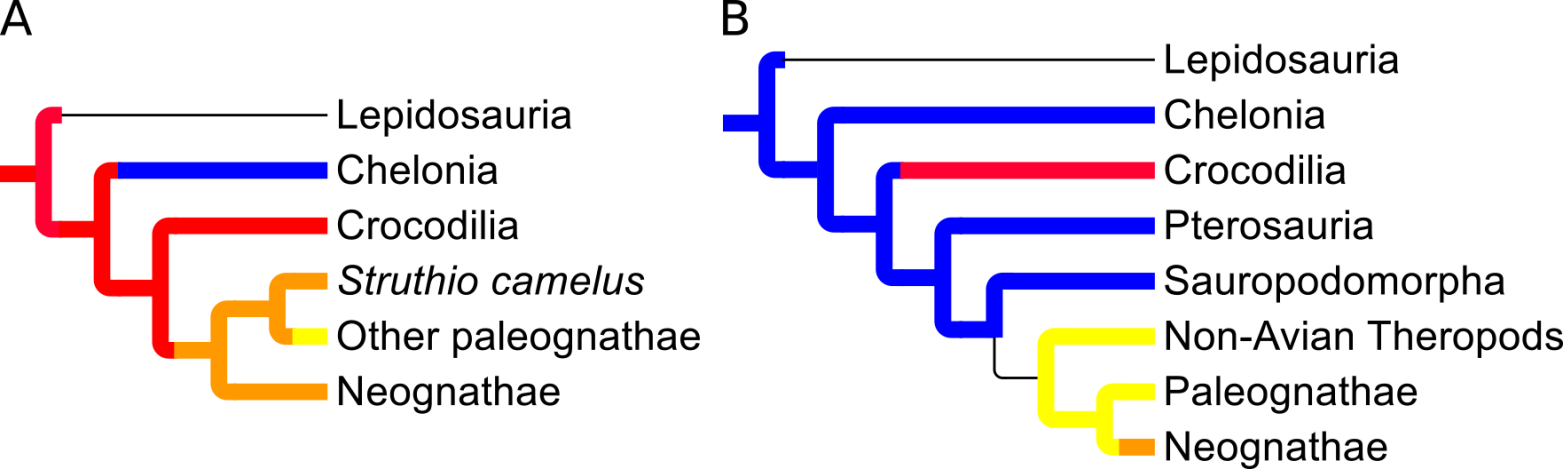
Summary of the most likely ancestral care strategies for major diapsid clades. Blue = no care, red = maternal care, orange = biparental care, yellow = paternal care, uncolored = heterogeneous/ambiguous. A = Extant taxa, B = Extinct and extant taxa.

Using this transition model, it is now possible to map the likelihoods of ancestral character states onto each node of our phylogeny, and hence make predictions regarding the ancestral care strategy of key clades based on the phylogeny of extant diapsids. Fig 1 shows a summary of the ancestral character likelihoods of several major clades, as predicted by this model using both datasets. Using only data from extant taxa, the root of the diapsid tree is approximately twice as likely to show maternal care than no care. Biparental care evolves in the clade including all extant birds (Neoaves), after their separation from the non-avian diapsids. Finally, paternal care evolves within the paleognaths, above the most primitive taxon, *S*. *camelus*. Note that even if *S*. *camelus* is coded as showing paternal care, Neoaves still show biparental care at their base. Predictions for every node in the phylogeny are presented in the supplementary information (S6 File). The overwhelming majority of the avian phylogeny is predicted to show biparental care, with the majority of transitions from this state occurring at shallow depth within the tree (S6 File).

Adding the extinct taxa to the phylogeny, and rerunning the ACE analysis using the transition likelihoods from Table 10 gives markedly different results towards the root of the tree (Fig 1B), highlighting the significance of the fossil data. The most likely care strategy at the root of the tree changes to no care, followed by the evolution of paternal care in derived theropod dinosaurs, and the evolution of biparental care from this, either at the base of Neoaves (likelihood = 0.5: This alters to 1 if *S*. *camelus* is coded as showing paternal care), or, if not, at the base of Neognatha. Patterns of care strategy evolution in smaller, more derived avian clades are unaffected (S7 File).

**Fig 2:**
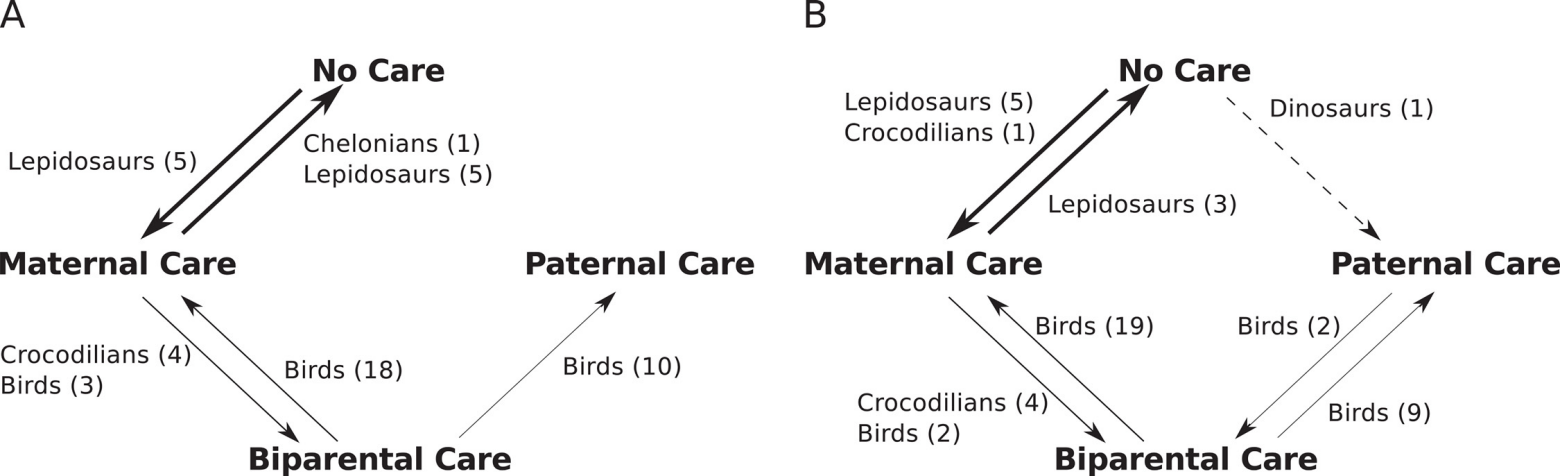
Most likely number of parental care character transitions from ancestral character estimation. Arrow thickness represents the assessed likelihood of each transition. Dotted = Very low likelihood. A = Extant only dataset, B = Extinct and extant taxa.

The character state reconstructions also provide an indication of the number of parental care state transitions that have occurred in each major clade, and the nature of those transitions (Fig 2). Interestingly, although allowed by the character transition model, no transitions from paternal care to either biparental or no care are observed in the extant-only dataset, and no transitions from paternal care to no care are observed in the extinct and extant dataset.

### Ancestral Character Estimation–Hatchling Precociality

Comparing the AIC values for the three candidate models (uniform, symmetrical, custom) shows that the symmetrical model, structured like Table 3B, was the best fitting model (AIC = 377.87 vs. 380.18 for the custom model and 406.73 for the uniform model, evidence ratios of 1, 3.17, and 1.85 × 10^6^, respectively). Character transition likelihoods are shown in Table 12.

**Table 12:** Character transition likelihoods for symmetrical hatchling precociality character transition model.

	1	2	3	4
0	0.0105	0.0000	0.0000	0.0000
1		0.0382	0.0013	0.0014
2			0.0751	0.0121
3				0.0135

0 = superprecocial, 1 = precocial, 2 = semi-precocial, 3 = subaltricial, 4 = altricial.

**Fig 3:**
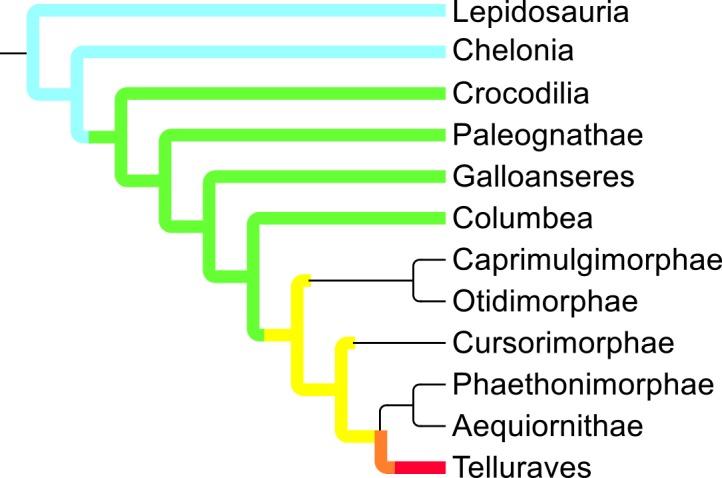
Summary of ancestral hatchling precociality states for extant taxa. Light blue = superprecocial, green = precocial, yellow = semiprecocial, orange = subaltricial, red = altricial, uncoloured = heterogeneous states.

**Fig 4:**
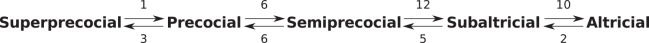
Most likely number of character transitions between different hatchling precociality states.

Mapping these onto the diapsid phylogeny shows a steady progression towards more altricial young as one moves through the roots of major diapsid clades towards and into derived birds (Fig 3), a pattern also illustrated by the number of transitions between each pair of states (Fig 4). Note that most of the reversions towards precociality occur in or near to terminal taxa, whereas many of the transitions towards altriciality occur deep within the phylogeny. Most major clades show marked homogeneity of hatchling precociality (S8 File). Three notable exceptions are the clades composed of 1) Caprimulgimorpha plus Otidimorphae, 2) Aequiornithia plus Phaethontimorphae (S9 File), and, to a lesser extent, 3) the Cursorimorphae. These clades show marked mutability in this character (for example, the clade containing *Balaeniceps rex*, *Scopus umbretta*, and *Pelecanus onocrotalus* reverts rapidly to precociality from a likely altricial ancestor), with little direct trend apparent.

Adding in the fossil taxa has a minimal influence on the patterns described here, so these analyses will not be reported in detail. Only the inclusion of the two hadrosaur taxa (assumed to be subaltricial) has any effect whatsoever, adding character transitions to the model (two from precocial to semiprecocial, and two from semiprecocial to subaltricial). These changes do not affect the character transition likelihoods in Table 12 or the overall pattern of hatchling precociality character transition in the diapsids.

### Ancestral Character Estimation–Polygamy

**Fig 5:**
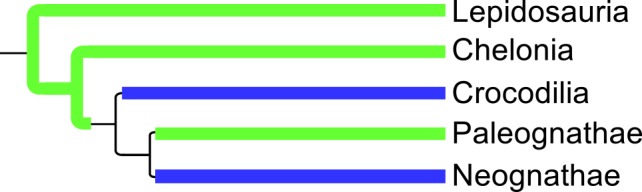
Summary of ancestral polygamy states for extant taxa. Green = both sexes polygamous, blue = socially monogamous, no colour = uncertain state.

When estimating the ancestral states of polygamy within the diapsids, the uniform transition model (with rate estimate of 0.0215) gave the best description of the observed character transition pattern (AIC = 531.91 vs. 538.05 for the symmetrical model and 602.29 for the custom model). Using this uniform model to predict the presence and type of polygamy (Fig 5) provides good support for both sexes showing polygamy among the earliest diapsids, transitioning to social monogamy somewhere between the origin of the archosaurs and the origin of the neognaths (both the ancestor of crocodilians and birds and the ancestor of paleognaths and neognaths have a 50% likelihood of showing each reproductive strategy). Both sexes show polygamy in most paleognath taxa. Monogamy completely dominates the ancestry of the neognathes, with a few isolated extant taxa and several small clades, Otididae (bustards), Rostratulidae (painted snipes) & Jacanidae (jacanas), Scolopacinae (woodcocks) & Gallinagininae (snipes), and Paradisaeidae (birds of paradise), showing any alternative strategies. The complete phylogeny with nodes labelled with character state likelihoods can be found in the Supplementary Information (S10 File).

**Fig 6:**
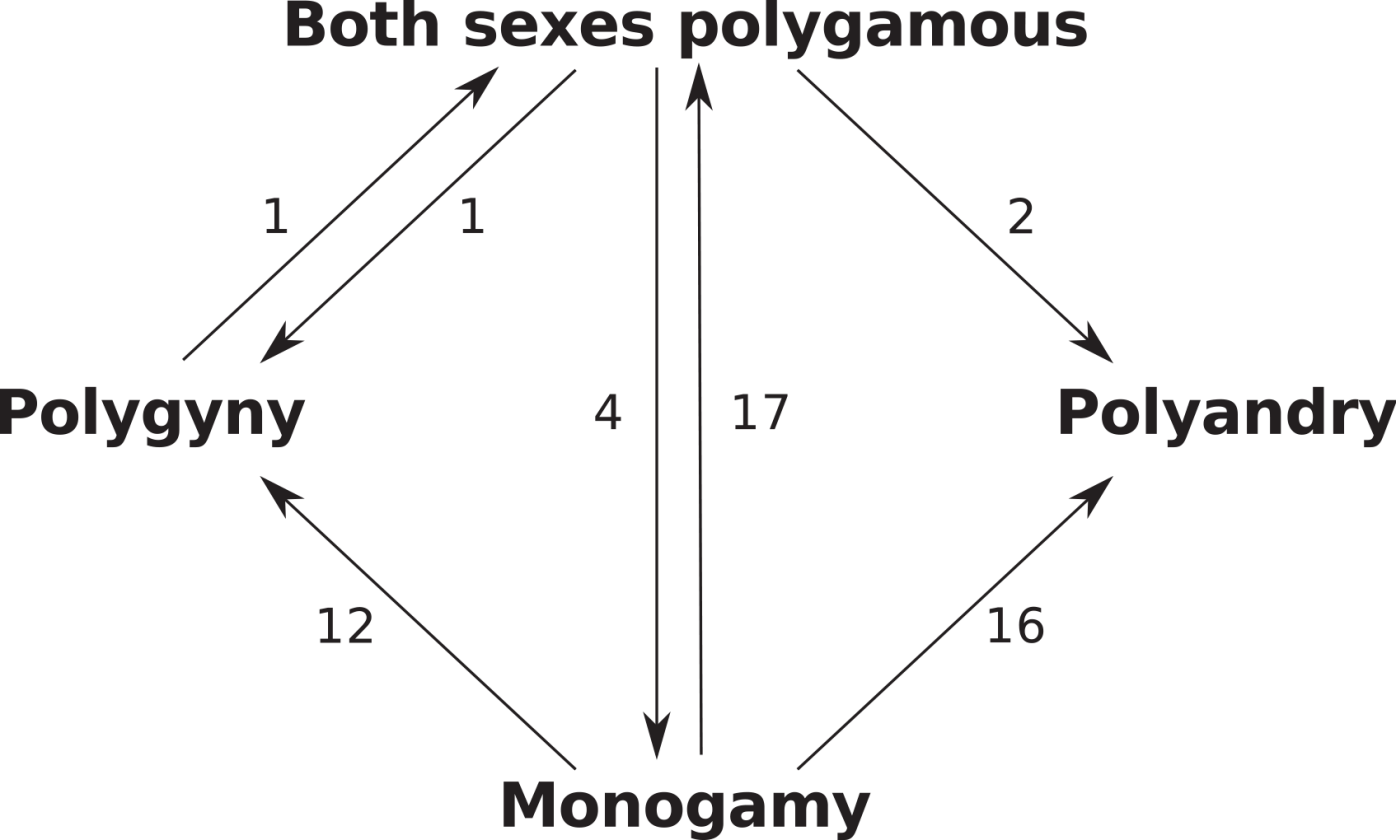
Most likely number of transitions between different polygamy character states.

Examining the most likely number of transitions between states of polygamy (Fig 6), it is clear that transitioning from monogamy to any other state is relatively common, whereas transitioning from polyandry (not observed) and polygyny (observed only once to both sexes polygamous) is very rare.

### Correlation of Care Strategy and Polygamy

A one-sided Fisher’s Exact Test shows a strong correlation of maternal care and polygyny, and paternal care and polyandry within our dataset (*p* < 0.001 in both cases, Table 13).

**Table 13:** Contingency tables of polygyny vs. maternal care and polyandry vs, paternal care among diapsids.

	Polygyny	No Polygyny		Polyandry	No Polyandry
Maternal Care	30	37	Paternal Care	26	18
No Maternal Care	86	318	No Paternal Care	95	332

## Discussion

### Influence of Reproductive Strategy on Clutch Volume

These analyses support past studies that have suggested some influence of both parental care sex and hatchling precociality on clutch volume in diapsids [14,47,107,119]. With our expanded dataset and non-linear analytical techniques which account for the degree of relatedness between studied taxa, we are able to show the influence of each parent care sex condition (no care, paternal, maternal, biparental) and degree of hatchling precociality on clutch volume. Of the care strategies, only biparental and maternal care cannot be distinguished statistically, although the absolute values of the model coefficients for each care sex agree with prior theoretical approaches [19] that suggest that clutch volume should scale negatively with degree of female contribution to parental care. Taxa showing paternal care show the largest clutch volumes, followed by those with biparental care, and finally taxa with maternal care. It should be noted, however, that the relative contribution of each sex in biparental taxa was not accounted for in these analyses, and so some error will be introduced for any taxa where care is unequally divided. This could lead to the conflation of biparental and maternal taxa if parental investment is skewed towards the female in many biparental taxa; as appears to be the case in most birds [10,120].

The results of these analyses mostly agree with the results of Varricchio et al. [47], who were able to differentiate the influence of each care sex using a simpler analytical technique, but contrast with the analyses of Birchard et al. [14] who were unable to differentiate maternal and paternal care using a binned taxonomic approach to account for the influence of relatedness. Varricchio et al. [47], however, differentiated the influence of avian maternal and avian biparental care from each other, but not from crocodile maternal care. It is likely that this crude binning of care strategies mixed the influence of phylogenetic relatedness and care strategy, producing the suggested, but apparently erroneous distinction between maternal and biparental care. An additional model adding higher taxon (~order) as a categorical variable to repeat the analyses of Birchard et al. [14] shows the same result as reported above for the care strategy variables, but with uniformly larger QIC values than the previously tested models. This indicates that the addition of these variables does not improve the explanatory power of the model; the influence of higher taxon on reproductive strategy, while present, is not of major importance in determining clutch volume when other factors have been accounted for.

The subset GEE analysis examining the influence of biparental and paternal care on only Charadriiformes (testing the hypothesis of Birchard et al. [14]) shows that this clade is an exception to the general pattern established for diapsids: Charadriiformes showing no influence of parental care on clutch volume. In addition, in this small sample, there appears to be little influence of precociality on clutch volume. These results, those of Birchard et al. [14], and the observation that Charadriiformes are one of the few avian groups where reverse size dimorphism has been shown to lead to markedly smaller than expected eggs [120–121], indicates that a closer examination of the reproductive ecology of this clade could be fruitful.

The addition of the extra variable separating the megapodes from the remainder of our analyzed taxa shows the positive influence on clutch volume suggested by Watson et al. [51], but produces a model with a larger QIC (39.30) than our best-fit model. This modification does not significantly change the nature and magnitude of influence of any of the other reproductive variables. As so few megapodes are represented in this dataset, their anomalous reproductive strategy is not markedly affecting the results of theses analyses.

Clutch volumes decrease slightly as neonates become more altricial. This decrease is expected due to the necessarily increased parental investment in post-hatching care for such neonates. Our data cannot differentiate whether this pattern represents a direct cost (limited parental resources available for food gathering: [120,122]), or an indirect cost (increased investment in young reduces parental reproductive fitness in the long term: [123]).

Surprisingly, the presence of either polyandry or polygyny in a taxon does not appear to significantly affect clutch volume. This could indicate that nest/clutch abandonment (which has been hypothesized to lead to uniparental care by one sex and polygamy by the other [52]) will only occur when the costs of abandonment to the first clutch are relatively low.

Applying these models to an expanded range of dinosaur taxa, including those of Varricchio et al. [47], increases support for paternal care in derived theropods (Table 7). Further support for this hypothesis comes from the low annual breeding frequency of theropods, as suggested by Werner and Griebeler [49]. The presence or absence of prominent sexual dimorphism in any of the four derived theropods would provide an additional test of our paternal care hypothesis. In birds, the non-caring sex often, although not always, displays prominent, sexually selected ornamentation/behavior [124–125], so the presence of dimorphism in fossil taxa could indicate either maternal or paternal care. Prominent sexual dimorphism has been hypothesized in some Cretaceous birds (*Confuciusornis sanctus* [126]), with the females potentially displaying the prominent ornamentation, which would provide some support for paternal care in this species. Given the likelihoods of the theropod dinosaurs *Troodon*, *Byronosaurus*, *Machairasaurus*, and *Citipati* showing paternal care (calculated earlier), we would predict that these taxa would be sexually dimorphic, and that the females should show the prominent ornamentation or behavior. In the case of size dimorphism, these results would suggest that the females of these theropod taxa should be the larger sex.

No parental care strategy is favored over any other in hadrosaurs, although some current data suggests maternal care in *Maiasaura* [1,31]. This discrepancy may occur because parental care strategy varies among the hadrosaurs [77]. Werner and Griebeler [49] found some support for such variation, suggesting that the lambeosaurine hadrosaur was more likely than *Maiasaura* to show extended parental care based on large egg size and smaller and less frequent clutches.

Reisz et al. [73] used the models of Varricchio et al. [47] to suggest that *Massospondylus* had maternal care. However, the model choices of Varricchio et al. [47] were predicated on the independent evidence of parental care in derived non-avian theropods, i.e. occurrences of adults with egg clutches, and thus did not include a “no care” model. Results of the present study, which allows for the possibility of the full range of care strategies, contradict the conclusion of Reisz et al. [73], and support that of Werner and Griebeler [49], suggesting that it is most likely that all sauropodomorphs for which we have data had no parental care.

### Ancestral Character Estimation

This study provides the most complete picture to date of the evolutionary patterns of reproductive strategies across the diapsids [7], particularly so with regards to the non-avian diapsids, and is the first to consider these data in the light of the most recent molecular and composite molecular-morphological phylogenies that have significantly modified our understanding of diapsid relationships [103]. It should be noted, however, that while the most of the major lepidosaurian and chelonian sub-clades are represented in this analysis, lower level coverage is nowhere near as complete as among the birds, so all the patterns discussed below are likely simplifications of the actual evolutionary histories of these non-avian groups. Similarly, data for extinct taxa are extremely limited. Additionally, the accuracy of the chosen phylogeny can strongly impact the results of an ACE analysis. While we have used the most up-to-date phylogenies available, it is possible that some of the clades that show significant heterogeneity of reproductive strategies appear this way due to inaccurate phylogenies, rather than evolutionary change.

#### Patterns of parental care strategy evolution

Before discussing our hypothesized pattern of parental care strategy evolution, it is necessary to decide whether the inclusion of the limited number of extinct diapsid taxa for which we have data is valid. The inclusion of the extinct taxa dramatically changes the ancestral diapsid parental care strategy, from maternal care without the extinct taxa to no care when they are included. This, in turn, affects the pattern of care strategy transitions at the base of the major diapsid clades (Fig 1).

Despite the limited available care strategy data, excluding the extinct archosaurs from our analyses leaves an ~150 million year gap between the last common ancestor of the crocodilians and birds, and the basalmost members of each of those clades. This gap represents an interval of greater than 50% of the duration of the entire existence of the diapsids. Given the amount of evolutionary change that can occur within such an extensive period, it is exceptionally hard to justify focusing such an analysis solely on extant taxa. Additionally, there is evidence for a range of parental care strategies among the extinct archosaur clades [1,4,31], suggesting that there has been significant evolutionary change during this period. As such we will limit our discussion to the results derived from analysis of our combined extinct-extant dataset. Nevertheless, the following discussion should be considered preliminary until independent data exist to better constrain a much greater number of extinct diapsid reproductive strategies.

The evolution of parental care amongst diapsids appears much more constrained than in other vertebrate groups, namely fish and anurans where this has been studied extensively [7,127]. In all three groups, the ancestral parental care strategy is no care with all three forms of parental care evolving from no care. In fish and anurans, transitions between each care strategy (paternal, maternal and biparental) once evolved are possible, but rare [7]. In diapsids we see a different pattern (Fig 2): Most evolutionary changes occur along a simple chain of transitions—No care <-> maternal care <-> biparental care -> paternal care. This could indicate a shift in the costs and benefits of nest abandonment by either parent in diapsids [52]. Character transition frequencies among the diapsids occur in approximately the same proportions as have been observed in previous, less-extensive analyses of birds [127] and the calculated character transition likelihoods (Table 11) also reflect this pattern. The exception to the pattern of transition along a chain of character states is the single shift from no care directly to paternal care within dinosaurs. This may reflect a unique evolutionary event, missing data for the extinct taxa highlighted earlier, or a linear series of transitions along the character state chain occurring between two nodes of this phylogeny that are not captured by the analysis.

Once a transition in parental care state has been made, there is often a significant degree of evolutionary inertia as reversions or transitions to another novel state are uncommon. This suggests there is a significant evolutionary cost to changing parental care strategy, perhaps unsurprisingly for such a critical biological function. This is particularly true for the loss of parental care, which occurs very infrequently in our dataset. This is in concurrence with parental care theory, as, once evolved, parental care tends to become rapidly complex, involving a range of different traits and integration between parental behavior and offspring development [128]. The loss of such a complex web of interacting behaviors and phenotypes is rare [129]. The exception to this general pattern appears to be in the lepidosaurs, where transitions from maternal care to and from no care are relatively common. While this may reflect a true evolutionary characteristic of the lepidosaurs, part of this pattern is likely attributable to the focus of our dataset on taxa that show parental care, rather than the majority that do not. We hypothesize that with the inclusion of a greater number of lepidosaur taxa, these transitions will become relatively less common, and that the pattern of character transition will appear more like that seen in birds.

These analyses suggest that the basal-most diapsids did not show any parental care. This is in agreement with prior expectations, perhaps slightly surprisingly, given the switching of chelonians and lepidosaurs at the base of the phylogeny. As chelonians are almost entirely lacking in parental care, and that they have traditionally been placed as the most primitive diapsids, no care was assumed to be the primitive condition among the diapsids. Even with lepidosaurs placed as the most basal diapsids, no care is retained as the likely basal condition for the crown group diapsids.

There has been significant debate as to the ancestral parental care strategy exhibited by both birds and dinosaurs [6,14,19,24,47], with debate focusing on the salience of paleontological data from dinosaurs to the crown group birds. These analyses support the assertion that paternal care was the primitive condition in birds [24,47]. The traditional view of biparental care as ancestral for crown group birds, supported by the analyses of Tullberg et al. [6] and Birchard et al. [14], appears contingent on the absence of data from extinct taxa. Betancur-R et al. [116] show how similar exclusions have led to misinterpretations of evolutionary patterns in other groups, as the extinction of taxa lying between extant lineages obfuscates the true pattern of character acquisition.

#### Patterns of evolution of precociality

Precociality shows a linear progression in the diapsids from superprecocial at the base of the clade to altricial amongst core land birds (Telluraves). This progression is reflected in the calculated character transition likelihoods, where by far the most likely transitions are from one level of precociality to that immediately above or below. We consider it likely that many of the modeled transitions that skip levels of precociality (i.e. precocial to subaltricial, or semiprecocial to altricial) are in fact caused by misclassification of taxon precociality, rather than representing accurate evolutionary patterns. Determination of hatching state depends upon a suite of characters and any one taxon may exhibit a mosaic of more altricial or precocial states across these characters. Degree of precociality should actually be considered to be a continuous character [130] that we have simply modeled as discrete and ordinal for analytical purposes. Such misclassification may also inflate the number of evolutionary transitions (and hence the character transition likelihoods) between some precociality categories (i.e. semiprecocial and subaltricial).

Given these caveats, however, it appears that the transitions to and from the end members of the spectrum (superprecocial and altricial) are the most evolutionarily unlikely, but that most states are conservative once evolved. As noted by Ricklefs and Starck [130], hatchling precociality tends to be similar amongst members of the same higher taxon. A noteworthy difference from previous phylogenetic studies of hatchling precociality is the uniform, and almost entirely unidirectional pattern of precociality state transition as taxa become more derived (Fig 3). Very few transitions are observed from more altricial to less altricial states, and these are almost all in the most terminal of taxa. This pattern has not been obvious in previous examinations of this topic due to the recent major changes in our understanding of diapsid, and particularly bird, phylogeny [103–105].

Significantly, this character state pattern suggests that there is a consistent selection pressure towards more altricial young in the major clades of diapsids, in contrast to some prior assertions [130]. The consistency of precociality state within many clades, as noted before, suggests that changes in precociality are evolutionarily costly, but advantageous in most ecological situations once achieved. Once taxa have evolved away from superprecociality it is exceptionally rare to return to that condition. Similarly, once taxa have evolved altricial young, reversion almost never occurs. This directional pattern can be seen along the root of the complete diapsid tree, as well as in several sub-clades. For example, this is the predominant pattern in the Aequiornithia plus Phaethontimorphae, one of the anomalous clades identified earlier, likely representing an independent expression of the overall drive towards altriciality. In contrast with the cases of polyandry and paternal care (which are evolutionary dead ends in this dataset), there are a limited number of transitions (2: Fig 4) to states of increased precociality from taxa that have evolved completely altricial young. This indicates some evolutionary plasticity in hatchling precociality, but the overwhelming trend remains towards greater altriciality.

Two other clades show significant variance from the overall unidirectional trend in precociality: the Cursorimorphae and the clade of Caprimulgimorpha plus Otidimorphae. The former represents a clade only recently recognized uniting Gruiformes and Charadriiformes [104]. There is a single reversion to precociality from semiprecociality at the base of the Charadriiformes, after which character states are relatively stable. In the Caprimulgimorpha plus Otidimorphae, the pattern of precociality is much more irregular, with multiple lineages transitioning in both directions on the precociality spectrum. However, within both clades the major shift toward more precocial offspring parallels changes from arboreal to ground nesting. Within otidimorphs this occurs between cuckoos and turacos versus bustards, and in caprimulgimorphs, from potoos, oilbirds, and frogmouths to nightjars.

Our ACE analysis places dinosaurs and pterosaurs unambiguously as primitively having precocial offspring, supporting previous fossil-based studies [32,91–96]. The transition to more altricial neonates in hadrosaur dinosaurs, e.g. *Maiasaura* [131], would be consistent with the overall pattern observed in this study of hatchling condition transitions.

#### Patterns of evolution of polygamy

More than 90% of the taxa in our dataset show the same polygamy state in both sexes (i.e. both sexes are polygamous or both sexes are monogamous). Polygynous or polyandrous species are rare, and are exclusively found in single derived species or small clades. As with both parental care strategy and hatchling precociality, the type of mating system is a relatively conservative trait among the diapsids; most clades are dominated by one character state, and change tends to occur only towards the tips of the phylogeny. This reinforces the hypothesis that changes in reproductive strategy are associated with significant fitness costs, and so are likely to be rare wherever observed. Interestingly given the observed pattern of character transitions (Fig 6), the best-fit character transition model was the uniform model (i.e. all transitions equally likely). As with paternal care, polyandry appears to be an evolutionary dead end, no taxa transition away from this condition after evolving it. Similarly, polygyny is very uncommonly lost once evolved; only once in the entire studied phylogeny. Both the correlation of paternal care and polyandry and that of maternal care with polygyny are supported by our data, providing support for the control of care strategy by breeding density [52]. Furthermore, once the strategies have evolved, both polyandry and paternal care are extremely stable in our analysis, again pointing to a link between these reproductive strategies.

As the nodes at the base of both Archosauria and Aves are equally likely to show social monogamy or polygamy of both sexes, it is not possible to infer the ancestral state of dinosaurs or pterosaurs from these data. The relationships demonstrated above, however, would suggest that the four taxa of theropod dinosaurs exhibiting paternal care should show low breeding density. This is consistent with their roles within Cretaceous ecosystems as carnivorous taxa. However, this is at odds with the density of *Troodon formosus* nests present at two of the prominent nesting sites where they have been recovered (Egg Mountain and Egg Island: [1]), although the amount of time-averaging present in those deposits is unclear.

### Summary/Coevolution of Reproductive Ecological Strategies

**Fig 7:**
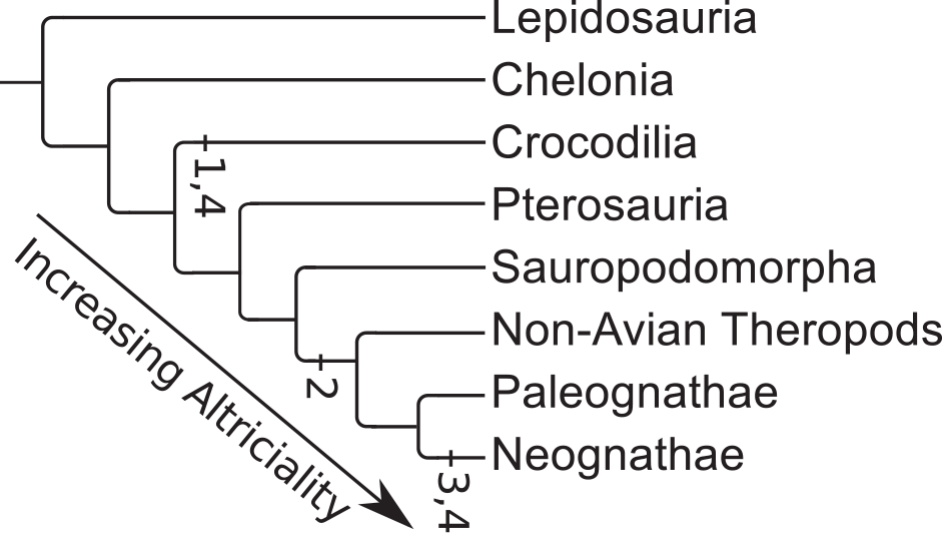
Summary of diapsid reproductive ecology evolution. The root of the tree shows no care, superprecocial offspring and polygamy of both sexes. 1 = transition to maternal care, 2 = transition to paternal care, 3 = transition to biparental care, 4 = transition to social monogamy.

Fig 7 summarizes the major changes in all of the reproductive strategies in the diapsids predicted by our analyses. While the transitions associated with increasing altriciality, and the acquisition of paternal care do not appear directly correlated with any of the other studied reproductive strategies, the transition to social monogamy (in major clades, at least) appears correlated with the evolution of a female role in parental care (either alone or as part of a biparental unit). This observation could have major evolutionary significance if a causal link can be suggested between the participation of the female in parental care and the evolution of social monogamy.

## Conclusions

These analyses represent the most comprehensive and up-to-date examination of the evolutionary pattern of diapsid reproductive strategies. The use of updated phylogenies and the analyses of multiple reproductive stretegies independently allow these analyses to reconcile some previous controversies of the evolution of parental care strategy, mating system and hatchling precociality.

In diapsids there is a strong relationship between parental care strategy and clutch volume, and between hatchling precociality and clutch volume, accounting for the influence of body mass. Clutch volumes increase as degree of paternal involvement in a clutch increases, and decrease as taxa become more altricial. Based on their clutch volumes, sauropodomorph dinosaurs likely did not care for their eggs, whereas derived non-avian theropod dinosaurs such as troodontids and oviraptorids likely showed paternal care. Hadrosaurs parental care cannot be determined.

The evolutionary transitions of parental care in diapsids occur almost entirely along a linear pathway from no care to maternal care to biparental care to paternal care. Basal diapsids show no care, with transitions to maternal care in crocodilians, paternal care in derived theropod dinosaurs, and biparental care in crown group neognaths. Basal dinosaurs showed no parental care.

Diapsid clades show a tendency to evolve towards more altricial young, albeit with a significant evolutionary cost to making changes to this reproductive strategy. Basal dinosaurs and pterosaurs had precocial young. Basal diapsids showed polygamy in both sexes, but evolution to social monogamy is relatively common. Polygyny and, particularly, polyandry are very stable states once evolved. Polyandry appears to correlate with paternal care, and polygyny with maternal care. Interestingly, the evolution of a female role in parental care occurs synchronously with the evolution of social monogamy in major diapsid clades.

## Supporting Information

S1 File Complete dataset used for GEE and ACE analyses. (PDF)

S2 File References used to compile reproductive ecology data. (PDF)

S3 File References used to produce composite phylogeny. (PDF)

S4 File The complete phylogeny used in these analyses including both extant and extinct taxa. For analyses where data for extinct taxa was not available, these were pruned from the tree.
(PDF)

S5 File Complete parental care strategy model comparison data for derived theropods, sauropodomorphs and hadrosaurs. (PDF)

S6 File Ancestral character estimation of parental care strategy in diapsids using only extant data. The proportions of colors in each the bars at each node of the phylogeny represents the likelihood of each care strategy at that node. Blue = no care, red = maternal care, orange = biparental care, yellow = paternal care.
(PDF)

S7 File Ancestral character estimation of parental care strategy in diapsids using complete dataset (extinct and extant taxa). The proportions of colors in each the bars at each node of the phylogeny represents the likelihood of each care strategy at that node. Blue = no care, red = maternal care, orange = biparental care, yellow = paternal care.
(PDF)

S8 File Ancestral character estimation of hatchling precociality state in diapsids. The proportions of colors in each the bars at each node of the phylogeny represents the likelihood of each care strategy at that node. Blue = no care, red = maternal care, orange = biparental care, yellow = paternal care. Light blue = superprecocial, green = precocial, yellow = semiprecocial, orange = subaltricial, red = altricial.
(PDF)

S9 File Clades demonstrating marked heterogeneity in hatchling precociality. A–Caprimulgimorpha + Otidimorphae, B–Aequiornithia + Phaethontimorphae. Green = precocial, yellow = semiprecocial, orange = subaltricial, red = altricial. The proportion of the colored bar at each node represents the likelihood of each precociality state at that node.
(PDF)

S10 File Ancestral character estimation of polygamy in diapsids. The proportions of colors in each the bars at each node of the phylogeny represents the likelihood of each care strategy at that node. Green = both sexes polygamous, blue = socially monogamous.
(PDF)
