# Supplementary material for: The Evolution of Diapsid Reproductive Strategy with Inferences about Extinct Taxa
Source: PLoS One. 2016 Jul 8;11(7):e0158496. doi: 10.1371/journal.pone.0158496 (PMC4938435; doi:10.1371/journal.pone.0158496)
Supplement: S1 File — (PDF) [file pone.0158496.s001.pdf]

Species	Higher Taxon	Family	Body Mass (kg)	Clutch Volume (mm3)	Hatchling Precociality	Male Polygamy	Female Polygamy	Paternal Care (No = 0, Yes = 1)	Maternal Care (No = 0, Yes = 1)	Biiparental Care (No = 0, Yes = 1)	No Care (No = 0, Yes = 1)
Sphenodon_punctatus	Sphenodontian	Sphenodontidae	0.366	44645	0	1	1	0	1	0	0
Oedura_ocellata	Lacertilian	Gekkonidae	0.0062	2144	0	1	1	0	0	0	1
Oedura_castelnaui	Lacertilian	Gekkonidae	0.0141	2695	0	1	1	0	0	0	1
Plestiodon_chinensis	Lacertilian	Scincidae	0.021	10570	0	1	1	0	1	0	0
Plestiodon_fasciatus	Lacertilian	Scincidae	0.0054	3400	0	1	1	0	1	0	0
Podarcis_bocagei	Lacertilian	Lacertidae	0.0025	972	0	1	1	0	0	0	1
Varanus_komodoensis	Lacertilian	Varanidae	70	2425203	0	1	1	0	0	0	1
Gambelia_wislizenii	Lacertilian	Crotaphytidae	0.0364	13181.88	0	1	1	0	0	0	1
Sceloporus_undulatus	Lacertilian	Phrynosomatidae	0.014	3840	0	1	1	0	0	0	1
Sauromalus_varius	Lacertilian	Iguanidae	0.171	63220	0	1	1	0	1	0	0
Iguana_iguana	Lacertilian	Iguanidae	0.575	291000	0	1	0	0	1	0	0
Cyclura_pinguis	Lacertilian	Iguanidae	3.021	783860	0	1	1	0	0	0	1
Cyclura_carinata	Lacertilian	Iguanidae	0.476	122080	0	1	1	0	1	0	0
Cyclura_collei	Lacertilian	Iguanidae	2.27	765400	0	1	1	0	0	0	1
Cyclura_cychlura	Lacertilian	Iguanidae	1.336	197294	0	1	1	0	1	0	0
Cyclura_rileyi	Lacertilian	Iguanidae	0.567	107900	0	1	1	0	0	0	1
Cyclura_stejnegeri	Lacertilian	Iguanidae	4.7	1308000	0	1	1	0	0	0	1
Python_regius	Serpent	Pythonidae	1.52	382680	0	1	1	0	1	0	0
Python_sebae	Serpent	Pythonidae	11.5	4774000	0	1	1	0	1	0	0
Python_molurus	Serpent	Pythonidae	29	7909120	0	1	1	0	1	0	0
Python_reticulatus	Serpent	Pythonidae	26	6592000	0	1	1	0	1	0	0
Liasis_fuscus	Serpent	Pythonidae	2.2	852798	0	1	1	0	1	0	0
Morelia_amesthistina	Serpent	Pythonidae	3	763000	0	1	1	0	1	0	0
Morelia_spilota	Serpent	Pythonidae	2.5	872770	0	1	1	0	1	0	0
Antaresia_childreni	Serpent	Pythonidae	0.6	124300	0	1	1	0	1	0	0
Micrurus_fulvius	Serpent	Elapidae	0.0338	17570	0	1	1	0	1	0	0
Pseudechis_butleri	Serpent	Elapidae	0.425	168060	0	1	1	0	1	0	0
Naja_melanoleuca	Serpent	Elapidae	1	396264	0	1	1	0	1	0	0
Daidophis_punctatus_regalis	Serpent	Colubridae	0.037	8832	0	1	1	0	0	0	1
Farancia_abacura	Serpent	Colubridae	0.147	51870	0	1	1	0	1	0	0
Hypsiglena_ochrorhyncha_ochrorhyncha	Serpent	Colubridae	0.03	10254	0	1	1	0	0	0	1
Tropidonophis_mairii	Serpent	Colubridae	0.1114	26482	0	1	1	0	1	0	0
Masticophis_bilineatus	Serpent	Colubridae	0.165	62586	0	1	1	0	0	0	1
Ptyas_mucosus	Serpent	Colubridae	0.658	362000	0	1	1	0	1	0	0
Elaphe_guttata	Serpent	Colubridae	0.123	63000	0	1	1	0	1	0	0
Rhinocheilus_lecontei	Serpent	Colubridae	0.055	16329	0	1	1	0	0	0	1
Lampropeltis_triangulum	Serpent	Colubridae	0.065	42950	0	1	1	0	0	0	1
Emydura_macquarii	Chelonian	Chelidae	1.95	64879.97	0	1	1	0	0	0	1
Pseudemys_scripta_troostii	Chelonian	Emydidae	1.122	84446.52	0	1	1	0	0	0	1
Chrysemys_rubriventris	Chelonian	Emydidae	3.477	129838.4	0	1	1	0	0	0	1
Chelydra_serpentina	Chelonian	Chelydridae	4.4	373196.35	0	1	1	0	0	0	1
Dermochelys_coriacea	Chelonian	Dermochelyidae	332.7	6495550	0	1	1	0	0	0	1
Chelonia_mydas	Chelonian	Cheloniidae	61.5	5166240	0	1	1	0	0	0	1
Natator_depressus	Chelonian	Cheloniidae	74.4	3717196.41	0	1	1	0	0	0	1
Eretmochelys_imbricata	Chelonian	Cheloniidae	50	3731000	0	1	1	0	0	0	1
Caretta_caretta	Chelonian	Cheloniidae	113	4068880	0	1	1	0	0	0	1
Lepidochelys_kempi	Chelonian	Cheloniidae	38.2	3388000	0	1	1	0	0	0	1
Lepidochelys_olivacea	Chelonian	Cheloniidae	38.1	3494820	0	1	1	0	0	0	1
Alligator_mississippiensis	Crocodilian	Alligatoridae	47.8	2540000	1	0	0	0	1	0	0
Paleosuchus_palpebrosus	Crocodilian	Alligatoridae	5.9	811000	1	0	0	0	1	0	0
Paleosuchus_trigonatus	Crocodilian	Alligatoridae	7.5	1150000	1	0	0	0	1	0	0
Caiman_yacare	Crocodilian	Alligatoridae	13	2230000	1	0	0	0	1	0	0
Caiman_latirostris	Crocodilian	Alligatoridae	14.6	2300000	1	0	0	0	0	1	0
Melanosuchus_niger	Crocodilian	Alligatoridae	82	4120000	1	0	0	0	1	0	0
Gavialis_gangeticus	Crocodilian	Gavialidae	147	7870000	1	1	0	0	1	0	0
Tomistoma_schlegelii	Crocodilian	Gavialidae	119	5380000	0	0	0	0	1	0	0
Osteolaemus_tetraspis	Crocodilian	Crocodylidae	18.8	556000	1	0	0	0	1	0	0
Crocodylus_porosus	Crocodilian	Crocodylidae	78.7	5420000	1	0	0	0	1	0	0
Crocodylus_palustris	Crocodilian	Crocodylidae	42.7	1950000	1	0	0	0	1	0	0
Crocodylus_moreletti	Crocodilian	Crocodylidae	31.7	2710000	1	0	0	0	1	0	0
Crocodylus_acutus	Crocodilian	Crocodylidae	76.7	2600000	1	0	0	0	0	1	0
Crocodylus_intermedius	Crocodilian	Crocodylidae	108	4970000	1	0	0	0	1	0	0
Crocodylus_johnstoni	Crocodilian	Crocodylidae	19.5	756000	1	0	0	0	1	0	0
Crocodylus_cataphractus	Crocodilian	Crocodylidae	50.5	2290000	1	0	0	0	1	0	0
Crocodylus_niloticus	Crocodilian	Crocodylidae	94.2	4310000	1	0	0	0	0	1	0
Crocodylus_novaeguineae	Crocodilian	Crocodylidae	39.9	2160000	1	0	0	0	0	1	0
Crocodylus_mindorensis	Crocodilian	Crocodylidae	36.9	997000	1	0	0	0	1	0	0
Pterosauria	Pterosaur	Pterosauria	NA	NA	1	NA	NA	0	0	0	1
Massospondylus	Dinosaur	Sauropodomorph	107	678000	1	NA	NA	0	0	0	1
Megaloolithus_patagonicus	Dinosaur	Sauropodomorph	5000	38500000	1	NA	NA	0	0	0	1
Megaloolithus_sirguei	Dinosaur	Sauropodomorph	10000	41500000	1	NA	NA	0	0	0	1
Citipati_osmolskae	Dinosaur	Theropod	79.23	8767000	1	NA	NA	1	0	0	0
Oviraptor_philoceratops	Dinosaur	Theropod	39	7140000	1	NA	NA	1	0	0	0

Troodon_formosus	Dinosaur	Theropod	51.4	7584000	1	NA	NA	1	0	0	0
Byronosaurus_jaffei	Dinosaur	Theropod	22.5	3240000	1	NA	NA	1	0	0	0
Struthio_camelus	Paleognaths	Struthionidae	111	22100000	1	1	1	0	0	1	0
Rhea_pennata	Paleognaths	Rheidae	20	9804900	1	1	1	1	0	0	0
Rhea_americana	Paleognaths	Rheidae	23	6600000	1	1	1	1	0	0	0
Dromaius_novaehollandiae	Paleognaths	Dromaiidae	34.2	5140000	1	0	1	1	0	0	0
Casuarius_casuarius	Paleognaths	Casuariidae	45.1	3220000	1	0	1	1	0	0	0
Apteryx_australis	Paleognaths	Apterygidae	2.33	617000	1	0	0	1	0	0	0
Apteryx_owenii	Paleognaths	Apterygidae	1.11	317000	1	0	0	1	0	0	0
Eudromia_elegans	Paleognaths	Tinamidae	0.704	263000	1	1	1	1	0	0	0
Rhynchotus_rufescens	Paleognaths	Tinamidae	0.845	265000	1	1	1	1	0	0	0
Nothura_maculosa	Paleognaths	Tinamidae	0.258	109000	1	1	1	1	0	0	0
Nothura_darwinii	Paleognaths	Tinamidae	0.246	122000	1	1	1	1	0	0	0
Nothoprocta_cinerascens	Paleognaths	Tinamidae	0.526	325000	1	1	1	1	0	0	0
Nothoprocta_ornata	Paleognaths	Tinamidae	0.622	252000	1	1	1	1	0	0	0
Nothoprocta_perdicaria	Paleognaths	Tinamidae	0.458	212000	1	1	1	1	0	0	0
Nothocercus_bonapartei	Paleognaths	Tinamidae	0.925	637000	1	1	1	1	0	0	0
Nothocercus_julius	Paleognaths	Tinamidae	0.2114	911040	0	1	0	1	0	0	0
Tinamus_major	Paleognaths	Tinamidae	1.03	307000	1	1	1	1	0	0	0
Tinamus_osgoodi	Paleognaths	Tinamidae	1.29	197000	1	1	1	1	0	0	0
Tinamus_tao	Paleognaths	Tinamidae	1.6	610000	1	1	1	1	0	0	0
Tinamus_solitarius	Paleognaths	Tinamidae	1.39	486000	1	1	1	1	0	0	0
Crypturellus_tataupa	Paleognaths	Tinamidae	0.22	93800	1	1	1	1	0	0	0
Crypturellus_parvirostris	Paleognaths	Tinamidae	0.22	74400	1	1	1	1	0	0	0
Crypturellus_soui	Paleognaths	Tinamidae	0.207	43600	1	1	1	1	0	0	0
Crypturellus_variegatus	Paleognaths	Tinamidae	0.317	30400	1	1	1	1	0	0	0
Crypturellus_strigulosus	Paleognaths	Tinamidae	0.431	136000	1	1	1	1	0	0	0
Crypturellus_undulatus	Paleognaths	Tinamidae	0.567	210000	1	1	1	1	0	0	0
Crypturellus_cinnamomeus	Paleognaths	Tinamidae	0.422	224000	1	1	1	1	0	0	0
Chauna_torquata	Galloanserae	Anhimidae	4.4	513000	1	0	0	0	0	1	0
Somateria_mollissima	Galloanserae	Anatidae	2.07	475000	1	0	0	0	1	0	0
Anas_platyrhynchos	Galloanserae	Anatidae	1.14	542000	1	0	0	0	1	0	0
Anser_anser	Galloanserae	Anatidae	3.31	797000	1	0	0	0	0	1	0
Cygnus_olor	Galloanserae	Anatidae	10.7	1750000	1	0	0	0	0	1	0
Cygnus_buccinator	Galloanserae	Anatidae	11.1	1443667	1	0	0	0	0	1	0
Alectura_lathami	Galloanserae	Megapodiidae	2.34	2580000	0	1	1	1	0	0	0
Leipoa_ocellata	Galloanserae	Megapodiidae	1.8	2710000	0	0	0	1	0	0	0
Crax_rubra	Galloanserae	Cracidae	4.13	358000	1	0	0	0	1	0	0
Acryllium_vulturinum	Galloanserae	Numididae	1.33	585000	1	0	0	0	0	1	0
Colinus_virginianus	Galloanserae	Odontophoridae	0.178	112000	1	0	0	0	0	1	0
Callipepla_gambelii	Galloanserae	Odontophoridae	0.166	104000	1	0	0	0	0	1	0
Argusianus_argus	Galloanserae	Phasianidae	1.99	134000	1	0	0	0	0	1	0
Gallus_gallus	Galloanserae	Phasianidae	0.78	158000	1	0	0	0	0	1	0
Francolinus_francolinus	Galloanserae	Phasianidae	0.453	140000	1	0	0	0	0	1	0
Alectoris_graeca	Galloanserae	Phasianidae	0.615	199000	1	0	0	0	0	1	0
Coturnix_chinensis	Galloanserae	Phasianidae	0.042	27600	1	0	0	0	0	1	0
Coturnix_coturnix	Galloanserae	Phasianidae	0.097	89800	1	1	1	0	0	1	0
Ammoperdix_griseogularis	Galloanserae	Phasianidae	0.203	87100	1	1	1	0	0	1	0
Perdicula_asiatica	Galloanserae	Phasianidae	0.07	31800	1	0	0	0	0	1	0
Centrocercus_urophasianus	Galloanserae	Tetraonidae	2.21	316000	1	1	0	0	1	0	0
Tympanuchus_cupido	Galloanserae	Tetraonidae	0.876	288000	1	1	0	0	1	0	0
Lagopus_lagopus	Galloanserae	Tetraonidae	0.571	208000	1	0	0	0	0	1	0
Falcipennis_canadensis	Galloanserae	Tetraonidae	0.474	208000	1	1	1	0	1	0	0
Tetrao_urogallus	Galloanserae	Tetraonidae	2.95	312000	1	1	1	0	1	0	0
Perdix_perdix	Galloanserae	Phasianidae	0.406	152000	1	1	1	0	0	1	0
Meleagris_gallopavo	Galloanserae	Meleagrididae	6.05	875000	1	1	0	0	1	0	0
Lophophorus_impejanus	Galloanserae	Phasianidae	2.08	371000	1	0	0	0	0	1	0
Phasianus_colchicus	Galloanserae	Phasianidae	1.14	3.00E+05	1	1	0	0	1	0	0
Pucrasia_macrolopha	Galloanserae	Phasianidae	1.06	218000	1	0	0	0	0	1	0
Podiceps_cristatus	Unnamed Clade	Podicipedidae	0.674	143000	1	0	0	0	0	1	0
Phoenicopterus_ruber	Unnamed Clade	Phoenicopteridae	3.07	134000	2	0	0	0	0	1	0
Columba_palumbus	Unnamed Clade	Columbidae	0.49	34800	4	0	0	0	0	1	0
Oena_capensis	Unnamed Clade	Columbidae	0.0406	5480	4	0	0	0	0	1	0
Monias_benschi	Unnamed Clade	Mestiornithidae	0.138	22900	1	1	1	1	0	0	0
Syrrhaptes_paradoxus	Unnamed Clade	Pteroclididae	0.257	56600	1	0	0	0	0	1	0
Pterocles_alchata	Unnamed Clade	Pteroclididae	0.285	55100	1	0	0	0	0	1	0
Pterocles_indicus	Unnamed Clade	Pteroclididae	0.179	29100	1	0	0	0	0	1	0
Nyctibius_griseus	Apodiforms, Caprimulgiforms and sister taxa	Nyctibiidae	0.173	16300	3	0	0	0	0	1	0
Steatornis_caripensis	Apodiforms, Caprimulgiforms and sister taxa	Steatornithidae	0.408	59500	4	0	0	0	0	1	0
Podargus_strigoides	Apodiforms, Caprimulgiforms and sister taxa	Podargidae	0.31	51100	3	0	0	0	0	1	0
Chordeiles_acutipennis	Apodiforms, Caprimulgiforms and sister taxa	Caprimulgidae	0.0485	10800	2	0	0	0	0	1	0
Selasphorus_rufus	Apodiforms, Caprimulgiforms and sister taxa	Trochilidae	0.0035	921	4	1	0	0	1	0	0
Hemiprocne_coronata	Land Birds	Hemiprocnidae	0.029	3610	2	0	0	0	0	1	0
Tachymarptis_melba	Apodiforms, Caprimulgiforms and sister taxa	Apodidae	0.103	12400	3	0	0	0	0	1	0

Caprimulgus_europaeus	Apodiforms, Caprimulgiforms and sister taxa	Caprimulgidae	0.067	15600	2	0	0	0	0	1	0
Macrodipteryx_vexillarius	Apodiforms, Caprimulgiforms and sister taxa	Caprimulgidae	0.0724	9640	2	1	1	0	0	1	0
Caprimulgus_indicus	Apodiforms, Caprimulgiforms and sister taxa	Caprimulgidae	0.0918	14800	2	0	0	0	0	1	0
Caprimulgus_macrurus	Apodiforms, Caprimulgiforms and sister taxa	Caprimulgidae	0.0662	14400	2	0	0	0	0	1	0
Crotophaga_ani	Core Gruiforms and Cuckoos	Cuculidae	0.112	50000	3	0	0	0	0	1	0
Coccyzus_americanus	Core Gruiforms and Cuckoos	Cuculidae	0.064	36000	4	0	0	0	0	1	0
Centropus_phasianinus	Core Gruiforms and Cuckoos	Cuculidae	0.37	57740	4	0	0	0	0	1	0
Centropus_sinensis	Core Gruiforms and Cuckoos	Cuculidae	0.283	54000	3	0	0	0	0	1	0
Centropus_grillii	Core Gruiforms and Cuckoos	Cuculidae	0.1	31415	4	0	1	1	0	0	0
Corythaeola_cristata	Water Birds and Musophagiforms	Musophagidae	0.965	67700	3	0	0	0	0	1	0
Eupodotis_ruficrista	Core Gruiforms and Cuckoos	Otididae	0.714	83200	1	1	1	0	1	0	0
Neotis_denhami	Core Gruiforms and Cuckoos	Otididae	4.83	225000	1	1	1	0	1	0	0
Tetrax_tetrax	Core Gruiforms and Cuckoos	Otididae	0.834	152000	1	1	1	0	1	0	0
Houbaropsis_bengalensis	Core Gruiforms and Cuckoos	Otididae	2.03	130000	1	1	1	0	1	0	0
Otis_tarda	Core Gruiforms and Cuckoos	Otididae	7.896	337264	1	0	0	0	1	0	0
Chlamydotis_undulata	Core Gruiforms and Cuckoos	Otididae	1.56	195000	1	1	1	0	1	0	0
Aramus_guarauna	Core Gruiforms and Cuckoos	Aramidae	1.08	352000	1	0	1	0	0	1	0
Balearica_pavonina	Core Gruiforms and Cuckoos	Gruidae	3.59	322000	3	0	0	0	0	1	0
Grus_antigone	Core Gruiforms and Cuckoos	Gruidae	5.96	396000	3	0	0	0	0	1	0
Grus_grus	Core Gruiforms and Cuckoos	Gruidae	5.5	382000	3	0	0	0	0	1	0
Gallirallus_striatus	Core Gruiforms and Cuckoos	Rallidae	0.116	83300	2	0	0	0	0	1	0
Fulica_atra	Core Gruiforms and Cuckoos	Rallidae	0.734	260000	2	0	0	0	0	1	0
Gallinula_chloropus	Core Gruiforms and Cuckoos	Rallidae	0.344	167000	2	0	1	0	0	1	0
Porphyrio_porphyrio	Core Gruiforms and Cuckoos	Rallidae	0.794	161000	2	1	1	0	0	1	0
Crex_crex	Core Gruiforms and Cuckoos	Rallidae	0.156	133000	1	0	0	0	1	0	0
Porzana_carolina	Core Gruiforms and Cuckoos	Rallidae	0.0748	76500	2	0	0	0	0	1	0
Aenigmatolimnas_marginalis	Core Gruiforms and Cuckoos	Rallidae	0.05	30990	1	0	1	1	0	0	0
Porzana_fusca	Core Gruiforms and Cuckoos	Rallidae	0.0576	62700	2	0	0	0	0	1	0
Porzana_parva	Core Gruiforms and Cuckoos	Rallidae	0.0497	50600	1	0	0	0	0	1	0
Burhinus_oedicnemus	Charadriforms	Burhinidae	0.459	93200	4	0	0	0	0	1	0
Chionis_alba	Charadriforms	Chionidae	0.677	98600	2	0	0	0	0	1	0
Pluvianus_aegyptius	Charadriforms	Glareolidae	0.082	22200	1	0	0	0	0	1	0
Vanellus_vanellus	Charadriforms	Charadriidae	0.219	102000	1	1	1	0	0	1	0
Charadrius_morinellus	Charadriforms	Charadriidae	0.12	58900	1	1	1	1	0	0	0
Charadrius_alexandrinus	Charadriforms	Charadriidae	0.0423	27600	1	0	0	0	0	1	0
Charadrius_hiaticula	Charadriforms	Charadriidae	0.0641	45800	1	0	0	0	0	1	0
Pluvialis_apricaria	Charadriforms	Charadriidae	0.214	116000	1	0	0	0	0	1	0
Haematopus_ostralegus	Charadriforms	Haematopodidae	0.526	161000	1	1	1	0	0	1	0
Ibidorhyncha_struthersii	Charadriforms	Ibidorhynchidae	0.294	135000	1	0	0	0	0	1	0
Himantopus_himantopus	Charadriforms	Recurvirostridae	0.161	85300	1	0	0	0	0	1	0
Recurvirostra_avosetta	Charadriforms	Recurvirostridae	0.304	119000	1	0	0	0	0	1	0
Pedionomus_torquatus	Charadriforms	Pedinomidae	0.0632	41800	1	0	1	0	0	1	0
Attagis_gayi	Charadriforms	Thinocoridae	0.311	105000	1	0	0	0	0	1	0
Thinocorus_orbignyanus	Charadriforms	Thinocoridae	0.115	52000	1	0	0	0	0	1	0
Rostratula_benghalensis	Charadriforms	Rostratulidae	0.121	41200	1	0	1	1	0	0	0
Actophilornis_africana	Charadriforms	Jacanidae	0.185	31642	1	1	1	1	0	0	0
Hydrophasianus_chirurgus	Charadriforms	Jacanidae	0.164	57900	1	0	1	1	0	0	0
Metopidius_indicus	Charadriforms	Jacanidae	0.155	44700	1	0	1	1	0	0	0
Jacana_jacana	Charadriforms	Jacanidae	0.15	38000	1	0	1	1	0	0	0
Jacana_spinosa	Charadriforms	Jacanidae	0.126	32103	1	0	1	1	0	0	0
Bartramia_longicauda	Charadriforms	Scolopacidae	0.159	104000	1	0	0	0	0	1	0
Numenius_arquata	Charadriforms	Scolopacidae	0.806	366000	1	0	0	0	0	1	0
Limosa_limosa	Charadriforms	Scolopacidae	0.291	197000	1	0	0	0	0	1	0
Scolopax_minor	Charadriforms	Scolopacidae	0.198	63000	1	1	0	0	0	1	0
Scolopax_rusticola	Charadriforms	Scolopacidae	0.31	115000	1	1	0	0	1	0	0
Coenocorypha_aucklandica	Charadriforms	Scolopacidae	0.109	37600	1	1	0	0	0	1	0
Gallinago_media	Charadriforms	Scolopacidae	0.171	76300	1	1	0	0	1	0	0
Arenaria_interpres	Charadriforms	Scolopacidae	0.136	69500	1	0	0	0	0	1	0
Philomachus_pugnax	Charadriforms	Scolopacidae	0.136	69900	1	1	0	0	1	0	0
Calidris_bairdii	Charadriforms	Scolopacidae	0.0411	31600	1	0	0	0	0	1	0
Phalaropus_tricolor	Charadriforms	Scolopacidae	0.06	32500	1	0	0	1	0	0	0
Phalaropus_fulicarius	Charadriforms	Scolopacidae	0.056	30100	1	0	1	1	0	0	0
Phalaropus_lobatus	Charadriforms	Scolopacidae	0.04	29000	1	0	1	1	0	0	0
Tringa_totanus	Charadriforms	Scolopacidae	0.129	85400	1	0	0	0	0	1	0
Actitis_hypoleucos	Charadriforms	Scolopacidae	0.048	47800	1	0	0	0	0	1	0
Actitis_macularius	Charadriforms	Scolopacidae	0.0404	37600	1	0	1	1	0	0	0
Turnix_sylvatica	Charadriforms	Turnicidae	0.054	18000	1	0	1	1	0	0	0
Turnix_varia	Charadriforms	Turnicidae	0.0925	26415	1	0	1	1	0	0	0
Rhinoptilus_africanus	Charadriforms	Glareolidae	0.0875	8810	1	0	0	0	0	1	0
Glareola_pratincola	Charadriforms	Glareolidae	0.0849	27800	1	0	0	0	0	1	0
Cursorius_cursor	Charadriforms	Glareolidae	0.138	21900	1	0	0	0	0	1	0
Stercorarius_longicaudus	Charadriforms	Stercorariidae	0.289	84700	1	0	0	0	0	1	0
Catharacta_skua	Charadriforms	Stercorariidae	1.34	168000	1	0	0	0	0	1	0
Fratercula_arctica	Charadriforms	Alcidae	0.652	56200	2	0	0	0	0	1	0

Cerorhinca_monocerata	Charadriforms	Alcidae	0.483	75500	2	0	0	0	0	1	0
Cepphus_grylle	Charadriforms	Alcidae	0.378	92300	2	0	0	0	0	1	0
Synthliboramphus_antiquus	Charadriforms	Alcidae	0.207	70400	1	0	0	0	0	1	0
Uria_aalge	Charadriforms	Alcidae	0.992	101000	2	0	0	0	0	1	0
Alle_alle	Charadriforms	Alcidae	0.181	26900	2	0	0	0	0	1	0
Alca_torda	Charadriforms	Alcidae	0.726	82200	2	0	0	0	0	1	0
Anous_stolidus	Charadriforms	Sternidae	0.178	33700	1	0	0	0	0	1	0
Gygis_alba	Charadriforms	Sternidae	0.111	20700	1	0	0	0	0	1	0
Gelochelidon_nilotica	Charadriforms	Sternidae	0.221	115000	1	0	0	0	0	1	0
Chlidonias_niger	Charadriforms	Sternidae	0.0653	32100	1	0	0	0	0	1	0
Chlidonias_hybridus	Charadriforms	Sternidae	0.0839	46800	1	0	0	0	0	1	0
Sterna_hirundo	Charadriforms	Sternidae	0.13	53400	1	0	0	0	0	1	0
Thalasseus_bengalensis	Charadriforms	Sternidae	0.209	35700	1	0	0	0	0	1	0
Rynchops_niger	Charadriforms	Rynchopidae	0.302	125000	1	0	0	0	0	1	0
Rissa_tridactyla	Charadriforms	Laridae	0.408	95400	1	0	0	0	0	1	0
Larus_ridibundus	Charadriforms	Laridae	0.284	149000	1	0	0	0	0	1	0
Rhodostethia_rosea	Charadriforms	Laridae	0.187	55000	1	0	0	0	0	1	0
Larus_minutus	Charadriforms	Laridae	0.118	81900	1	0	0	0	0	1	0
Larus_marinus	Charadriforms	Laridae	1.66	386000	1	0	0	0	0	1	0
Larus_hyperboreus	Charadriforms	Laridae	1.54	357000	1	0	0	0	0	1	0
Larus_argentatus	Charadriforms	Laridae	1.09	259000	1	0	0	0	0	1	0
Phaethon_aethereus	Unnamed Clade	Phaethontidae	0.75	51700	2	0	0	0	0	1	0
Rhynochetos_jubata	Apodiforms, Caprimulgiforms and sister taxa	Rhynochetidae	0.86	64900	2	0	0	0	0	1	0
Gavia_immer	Water Birds and Musophagiforms	Gaviidae	4.98	317000	1	0	0	0	0	1	0
Aptenodytes_forsteri	Water Birds and Musophagiforms	Spheniscidae	32.55	400945	3	0	0	0	0	1	0
Aptenodytes_patagonica	Water Birds and Musophagiforms	Spheniscidae	11.8	286000	2	0	0	0	0	1	0
Hydrobates_pelagicus	Water Birds and Musophagiforms	Hydrobatidae	0.0252	5890	3	0	0	0	0	1	0
Diomedea_exulans	Water Birds and Musophagiforms	Diomedeidae	7.05	447000	3	0	0	0	0	1	0
Diomedea_epomorpha	Water Birds and Musophagiforms	Diomedeidae	9	419811	2	0	0	0	0	1	0
Fulmarus_glacialis	Water Birds and Musophagiforms	Procellariidae	0.613	88800	3	0	0	0	0	1	0
Pelecanoides_urinatrix	Water Birds and Musophagiforms	Pelecanoididae	0.141	19100	3	0	0	0	0	1	0
Bulweria_bulwerii	Water Birds and Musophagiforms	Procellariidae	0.099	20200	3	0	0	0	0	1	0
Ciconia_ciconia	Water Birds and Musophagiforms	Ciconiidae	3.45	387000	2	0	0	0	0	1	0
Fregata_magnificens	Water Birds and Musophagiforms	Fregatidae	1.41	117000	2	0	0	0	0	1	0
Phalacrocorax_carbo	Water Birds and Musophagiforms	Phalacrocoracidae	2.57	181000	4	0	0	0	0	1	0
Sula_bassana	Water Birds and Musophagiforms	Sulidae	3	85700	2	0	0	0	0	1	0
Anhinga_anhinga	Water Birds and Musophagiforms	Anhingidae	1.24	153000	2	0	0	0	0	1	0
Eudocimus_ruber	Water Birds and Musophagiforms	Threskiornithidae	0.665	125000	3	0	0	0	0	1	0
Geronticus_eremita	Water Birds and Musophagiforms	Threskiornithidae	1.2	159000	3	0	0	0	0	1	0
Plegadis_falcinellus	Water Birds and Musophagiforms	Threskiornithidae	0.634	120000	3	0	0	0	0	1	0
Bostrychia_hagedash	Water Birds and Musophagiforms	Threskiornithidae	1.24	135000	3	0	0	0	0	1	0
Threskiornis_aethiopicus	Water Birds and Musophagiforms	Threskiornithidae	1.5	144000	3	0	0	0	0	1	0
Platalea_ajaja	Water Birds and Musophagiforms	Threskiornithidae	1.49	220000	3	0	0	0	0	1	0
Pelecanus_onocrotalus	Water Birds and Musophagiforms	Pelecanidae	9.52	316000	4	0	0	0	0	1	0
Scopus_umbretta	Water Birds and Musophagiforms	Scopidae	0.472	111000	2	0	0	0	0	1	0
Balaeniceps_rex	Water Birds and Musophagiforms	Balaenicipitidae	5.98	297000	1	0	0	0	0	1	0
Botaurus_stellaris	Water Birds and Musophagiforms	Ardeidae	1.33	183000	4	0	0	0	0	1	0
Ixobrychus_minutus	Water Birds and Musophagiforms	Ardeidae	0.101	85700	4	0	0	0	0	1	0
Ardeola_ralloides	Water Birds and Musophagiforms	Ardeidae	0.287	49500	4	0	0	0	0	1	0
Ardea_herodias	Water Birds and Musophagiforms	Ardeidae	2.3	272000	4	0	0	0	0	1	0
Bubulcus_ibis	Water Birds and Musophagiforms	Ardeidae	0.366	77500	4	0	0	0	0	1	0
Tyto_alba	Land Birds	Tytonidae	0.418	114000	3	0	0	0	0	1	0
Bubo_bubo	Land Birds	Strigidae	2.69	287000	3	0	0	0	0	1	0
Colius_striatus	Land Birds	Coliidae	0.0511	8750	4	0	0	0	0	1	0
Trogon_citreolus	Land Birds	Trogonidae	0.079	23100	4	0	0	0	0	1	0
Pharomachrus_mocinno	Land Birds	Trogonidae	0.203	25100	4	0	0	0	0	1	0
Aceros_nipalensis	Land Birds	Bucerotidae	2.39	84300	4	0	0	0	0	1	0
Phoeniculus_purpureus	Land Birds	Phoeniculidae	0.0743	13400	4	0	0	0	0	1	0
Upupa_epops	Land Birds	Upupidae	0.0671	26900	4	0	0	0	0	1	0
Dryocopus_martius	Land Birds	Picidae	0.321	51300	4	0	0	0	0	1	0
Galbula_ruficauda	Land Birds	Galbulidae	0.0265	9840	4	0	0	0	0	1	0
Chelidoptera_tenebrosa	Land Birds	Bucconidae	0.0359	7340	4	0	0	0	0	1	0
Merops_philippinus	Land Birds	Meropidae	0.034	27300	4	0	0	0	0	1	0
Coracias_benghalensis	Land Birds	Coraciidae	0.158	48300	4	0	0	0	0	1	0
Todus_todus	Land Birds	Todidae	0.0064	5040	4	0	0	0	0	1	0
Alcedo_atthis	Land Birds	Alcedinidae	0.0314	20100	4	0	0	0	0	1	0
Momotus_mexicanus	Land Birds	Momotidae	0.0757	25000	4	0	0	0	0	1	0
Cathartes_aura	Land Birds	Cathartidae	1.72	160000	3	0	0	0	0	1	0
Vultur_gryphus	Land Birds	Cathartidae	11.3	278000	3	0	0	0	0	1	0
Sarcorhamphus_papa	Land Birds	Cathartidae	3.4	187000	3	0	0	0	0	1	0
Coragyps_atratus	Land Birds	Cathartidae	1.9	191000	3	0	0	0	0	1	0
Gymnogyps_californicus	Land Birds	Cathartidae	8.45	258746	2	0	0	0	0	1	0
Sagittarius_serpentarius	Land Birds	Sagittariidae	4.02	335000	3	0	0	0	0	1	0
Pandion_haliaetus	Land Birds	Pandionidae	1.49	193000	3	1	0	0	0	1	0

Elanus_caeruleus	Land Birds	Accipitridae	0.261	74000	3	0	1	0	0	1	0
Pernis_apivorus	Land Birds	Accipitridae	0.758	89200	3	0	0	0	0	1	0
Polyboroides_typus	Land Birds	Accipitridae	0.638	103000	3	0	0	0	0	1	0
Neophron_percnopterus	Land Birds	Accipitridae	2.08	160000	3	0	0	0	0	1	0
Gypaetus_barbatus	Land Birds	Accipitridae	5.7	254000	3	0	0	0	0	1	0
Circaetus_gallicus	Land Birds	Accipitridae	1.7	126000	3	0	0	0	0	1	0
Spilornis_cheela	Land Birds	Accipitridae	0.628	94900	3	0	0	0	0	1	0
Gyps_fulvus	Land Birds	Accipitridae	7.44	232000	3	0	0	0	0	1	0
Torgos_tracheliotus	Land Birds	Accipitridae	6.97	227000	3	0	0	0	0	1	0
Aegypius_monachus	Land Birds	Accipitridae	9.63	211000	3	0	0	0	0	1	0
Aquila_chrysaetos	Land Birds	Accipitridae	4.26	271000	3	0	0	0	0	1	0
Haliaeetus_leucocephalus	Land Birds	Accipitridae	4.74	219000	3	0	0	0	0	1	0
Haliastur_indus	Land Birds	Accipitridae	0.529	109000	3	0	0	0	0	1	0
Milvus_milvus	Land Birds	Accipitridae	1.08	171000	3	0	0	0	0	1	0
Buteo_lagopus	Land Birds	Accipitridae	0.956	197000	3	0	0	0	0	1	0
Circus_pygargus	Land Birds	Accipitridae	0.316	139000	3	1	0	0	0	1	0
Accipiter_gentilis	Land Birds	Accipitridae	0.902	180000	3	0	0	0	0	1	0
Accipiter_nisus	Land Birds	Accipitridae	0.238	92200	3	0	0	0	0	1	0
Cariama_cristata	Land Birds	Cariamidae	1.4	143000	4	0	0	0	0	1	0
Polyborus_cheriway	Land Birds	Falconidae	0.894	187000	3	0	0	0	0	1	0
Polihierax_semitorquatus	Land Birds	Falconidae	0.0615	18700	3	0	1	0	0	1	0
Falco_rusticolus	Land Birds	Falconidae	1.46	272000	3	0	0	0	0	1	0
Falco_naumanni	Land Birds	Falconidae	0.153	64300	3	0	0	0	0	1	0
Falco_peregrinus	Land Birds	Falconidae	0.783	127000	3	0	0	0	0	1	0
Falco_vespertinus	Land Birds	Falconidae	0.153	57500	3	0	0	0	0	1	0
Psittacula_krameri	Land Birds	Psittacidae	0.117	36400	4	0	0	0	0	1	0
Pitta_nipalensis	Land Birds	Pittidae	0.124	31300	4	0	0	0	0	1	0
Serilophus_lunatus	Land Birds	Eurylaimidae	0.034	17700	4	0	0	0	0	1	0
Psarisomus_dalhousiae	Land Birds	Eurylaimidae	0.067	25900	4	0	0	0	0	1	0
Taraba_major	Land Birds	Thamnophilidae	0.0592	16000	4	0	0	0	0	1	0
Drymornis_bridgesii	Land Birds	Dendrocolaptidae	0.094	21100	4	0	0	0	0	1	0
Hylopezus_perspicillatus	Land Birds	Formicariidae	0.043	11100	4	0	0	0	0	1	0
Scytalopus_magellanicus	Land Birds	Rhinocryptidae	0.0136	6850	4	0	0	0	0	1	0
Manacus_manacus	Land Birds	Pipridae	0.0192	5020	4	0	0	0	1	0	0
Rupicola_rupicola	Land Birds	Cotingidae	0.195	43000	4	0	0	0	1	0	0
Phytotoma_rara	Land Birds	Cotingidae	0.047	12400	4	0	0	0	0	1	0
Elaenia_flavogaster	Land Birds	Tyrannidae	0.0248	4700	4	0	0	0	0	1	0
Myiarchus_crinitus	Land Birds	Tyrannidae	0.0321	16000	4	0	0	0	0	1	0
Tyrannus_melancholicus	Land Birds	Tyrannidae	0.0374	16500	4	0	0	0	0	1	0
Menura_novaehollandiae	Land Birds	Menuridae	0.98	58500	4	1	1	0	1	0	0
Sericulus_chrysocephalus	Land Birds	Ptilonorhynchidae	0.1	23900	4	1	0	0	1	0	0
Malurus_cyaneus	Land Birds	Maluridae	0.0101	4740	4	0	1	0	0	1	0
Anthornis_melanura	Land Birds	Meliphagidae	0.0272	12100	4	0	0	0	0	1	0
Prosthemadera_novaeseelandiae	Land Birds	Meliphagidae	0.107	25600	4	0	0	0	0	1	0
Creadion_carunculatus	Land Birds	Callaeatidae	0.075	17700	4	0	0	0	0	1	0
Pachycephala_pectoralis	Land Birds	Pachycephalidae	0.0303	7180	4	0	0	0	0	1	0
Vanga_curvirostris	Land Birds	Vangidae	0.0645	13100	4	0	0	0	0	1	0
Tchagra_senegala	Land Birds	Malaconotidae	0.0535	11000	4	0	0	0	0	1	0
Laniarius_ferrugineus	Land Birds	Malaconotidae	0.0468	9370	4	0	0	0	0	1	0
Rhipidura_albicollis	Land Birds	Rhipiduridae	0.0129	3640	4	0	0	0	0	1	0
Hypothymis_azurea	Land Birds	Monarchidae	0.0111	5280	4	0	0	0	0	1	0
Terpsiphone_paradisi	Land Birds	Monarchidae	0.0185	7690	4	0	0	0	0	1	0
Dicrurus_aeneas	Land Birds	Dicruridae	0.0265	12300	4	0	0	0	0	1	0
Dicrurus_hottentottus	Land Birds	Dicruridae	0.0792	22100	4	0	0	0	0	1	0
Dicrurus_macrocercus	Land Birds	Dicruridae	0.0483	15600	4	0	0	0	0	1	0
Dicrurus_adsimilis	Land Birds	Dicruridae	0.0403	9160	4	0	0	0	0	1	0
Manucodia_keraudrenii	Land Birds	Paradisaeidae	0.156	22000	4	0	0	0	0	1	0
Paradisaea_minor	Land Birds	Paradisaeidae	0.214	19300	4	1	0	0	1	0	0
Seleucides_melanoleuca	Land Birds	Paradisaeidae	0.175	14100	4	1	0	0	1	0	0
Ptiloris_paradiseus	Land Birds	Paradisaeidae	0.121	18900	4	1	0	0	1	0	0
Lophorina_superba	Land Birds	Paradisaeidae	0.077	7070	4	1	0	0	1	0	0
Lanius_collurio	Land Birds	Laniidae	0.0285	15800	4	0	0	0	0	1	0
Lanius_senator	Land Birds	Laniidae	0.036	19500	4	0	0	0	0	1	0
Pyrrhocorax_pyrrhocorax	Land Birds	Corvidae	0.278	55900	4	0	0	0	0	1	0
Perisoreus_infaustus	Land Birds	Corvidae	0.0844	32500	4	0	0	0	0	1	0
Cyanopica_cyana	Land Birds	Corvidae	0.096	38600	4	0	0	0	0	1	0
Cyanocorax_melanocyaneus	Land Birds	Corvidae	0.101	22400	4	0	0	0	0	1	0
Cyanocorax_sanblasiana	Land Birds	Corvidae	0.109	26900	4	0	0	0	0	1	0
Aphelocoma_californica	Land Birds	Corvidae	0.0862	20900	4	0	0	0	0	1	0
Cyanocitta_cristata	Land Birds	Corvidae	0.088	22400	4	0	0	0	0	1	0
Garrulus_glandarius	Land Birds	Corvidae	0.168	45700	4	0	0	0	0	1	0
Podoces_panderi	Land Birds	Corvidae	0.0911	21300	4	0	0	0	0	1	0
Pica_pica	Land Birds	Corvidae	0.218	64300	4	0	0	0	0	1	0
Nucifraga_caryocatactes	Land Birds	Corvidae	0.183	28300	4	0	0	0	0	1	0

Corvus_monedula	Land Birds	Corvidae	0.246	49500	4	0	0	0	0	1	0
Corvus_capensis	Land Birds	Corvidae	0.553	73100	4	0	0	0	0	1	0
Corvus_corax	Land Birds	Corvidae	0.941	148000	4	0	0	0	0	1	0
Parus_major	Land Birds	Paridae	0.0158	10800	4	0	0	0	0	1	0
Chersomanes_albofasciata	Land Birds	Alaudidae	0.0494	5880	4	0	0	0	0	1	0
Ammomanes_phoenicurus	Land Birds	Alaudidae	0.0256	7230	4	0	0	0	0	1	0
Calandrella_cinerea	Land Birds	Alaudidae	0.0237	8790	4	0	0	0	0	1	0
Eremophila_alpestris	Land Birds	Alaudidae	0.0335	11800	4	0	0	0	0	1	0
Galerida_cristata	Land Birds	Alaudidae	0.0428	12200	4	0	0	0	0	1	0
Alauda_arvensis	Land Birds	Alaudidae	0.0375	11200	4	0	0	0	0	1	0
Lullula_arborea	Land Birds	Alaudidae	0.0269	9010	4	0	0	0	0	1	0
Hirundo_rustica	Land Birds	Hirundinidae	0.018	8160	4	0	0	0	0	1	0
Andropadus_importunus	Land Birds	Pycnonotidae	0.0274	6010	4	0	0	0	0	1	0
Pycnonotus_cafer	Land Birds	Pycnonotidae	0.043	6740	4	0	0	0	0	1	0
Pycnonotus_jocosus	Land Birds	Pycnonotidae	0.0296	6590	4	0	0	0	0	1	0
Donacobius_atricapillus	Land Birds	Troglodytidae	0.0368	6630	4	0	0	0	0	1	0
Chamaea_fasciata	Land Birds	Sylviidae	0.0148	7400	4	0	0	0	0	1	0
Sylvia_hortensis	Land Birds	Sylviidae	0.0219	9650	4	0	0	0	0	1	0
Sylvia_borin	Land Birds	Sylviidae	0.0182	9650	4	0	0	0	0	1	0
Sylvia_atricapilla	Land Birds	Sylviidae	0.0167	10500	4	0	0	0	0	1	0
Cettia_cetti	Land Birds	Sylviidae	0.0134	6690	4	1	1	0	0	1	0
Aegithalos_caudatus	Land Birds	Aegithalidae	0.0086	7870	4	0	0	0	0	1	0
Leptopoecile_sophiae	Land Birds	Aegithalidae	0.0069	4470	4	0	0	0	0	1	0
Cisticola_juncidis	Land Birds	Cisticolidae	0.0069	5060	4	1	0	0	0	1	0
Orthotomus_sutorius	Land Birds	Cisticolidae	0.0075	3140	4	0	0	0	0	1	0
Prinia_inornata	Land Birds	Cisticolidae	0.007	4300	4	0	0	0	0	1	0
Prinia_subflava	Land Birds	Cisticolidae	0.0087	3540	4	0	0	0	0	1	0
Hippolais_caligata	Land Birds	Sylviidae	0.0089	4700	4	0	0	0	0	1	0
Acrocephalus_palustris	Land Birds	Sylviidae	0.0115	7370	4	0	0	0	0	1	0
Zosterops_palpebrosa	Land Birds	Zosteropidae	0.0086	3040	4	0	0	0	0	1	0
Pomatorhinus_schisticeps	Land Birds	Timaliidae	0.043	14500	4	0	0	0	0	1	0
Napothera_brevicaudata	Land Birds	Timaliidae	0.0195	9820	4	0	0	0	0	1	0
Turdoides_caudatus	Land Birds	Timaliidae	0.0397	10900	4	1	0	0	0	1	0
Liocichla_phoenicea	Land Birds	Timaliidae	0.049	11300	4	0	0	0	0	1	0
Promerops_cafer	Land Birds	Promeropidae	0.0349	7770	4	1	1	0	0	1	0
Cyanomitra_olivacea	Land Birds	Nectariniidae	0.0099	2640	4	1	0	0	0	1	0
Arachnothera_longirostra	Land Birds	Nectariniidae	0.0126	3100	4	0	0	0	0	1	0
Arachnothera_magna	Land Birds	Nectariniidae	0.0307	4930	4	0	0	0	0	1	0
Irena_puella	Land Birds	Irenidae	0.0649	11800	4	1	1	0	0	1	0
Prunella_modularis	Land Birds	Prunellidae	0.0203	8770	4	1	1	0	0	1	0
Passer_domesticus	Land Birds	Passeridae	0.026	9640	4	0	0	0	0	1	0
Montifringilla_nivalis	Land Birds	Passeridae	0.0369	15000	4	0	0	0	0	1	0
Motacilla_flava	Land Birds	Motacillidae	0.0177	10400	4	0	0	0	0	1	0
Motacilla_cinerea	Land Birds	Motacillidae	0.0172	9500	4	0	0	0	0	1	0
Macronyx_croceus	Land Birds	Motacillidae	0.0471	10200	4	0	0	0	0	1	0
Anthus_novaeseelandiae	Land Birds	Motacillidae	0.0241	14000	4	0	0	0	0	1	0
Anthus_trivialis	Land Birds	Motacillidae	0.0234	12300	4	0	0	0	0	1	0
Fringilla_coelebs	Land Birds	Fringillidae	0.024	9650	4	0	0	0	0	1	0
Fringilla_teydea	Land Birds	Fringillidae	0.0303	4700	4	0	0	0	0	1	0
Coccothraustes_coccothraustes	Land Birds	Fringillidae	0.0567	10200	4	0	0	0	0	1	0
Pinicola_enucleator	Land Birds	Fringillidae	0.0564	15300	4	0	0	0	0	1	0
Pyrrhula_pyrrhula	Land Birds	Corvidae	0.0244	9000	4	0	0	0	0	1	0
Carpodacus_erythrinus	Land Birds	Fringillidae	0.024	10200	4	0	0	0	0	1	0
Loxia_curvirostra	Land Birds	Fringillidae	0.0385	9220	4	0	0	0	0	1	0
Serinus_serinus	Land Birds	Fringillidae	0.0112	4550	4	0	0	0	0	1	0
Carduelis_carduelis	Land Birds	Fringillidae	0.016	6570	4	0	0	0	0	1	0
Calcarius_lapponicus	Land Birds	Emberizidae	0.0279	11800	4	0	0	0	0	1	0
Plectrophenax_nivalis	Land Birds	Emberizidae	0.0422	13500	4	0	0	0	0	1	0
Cardinalis_cardinalis	Land Birds	Cardinalidae	0.0427	13400	4	0	0	0	0	1	0
Spiza_americana	Land Birds	Cardinalidae	0.0263	10000	4	1	0	0	1	0	0
Dendroica_petechia	Land Birds	Parulidae	0.0096	6400	4	0	0	0	0	1	0
Protonotaria_citrea	Land Birds	Parulidae	0.0143	9920	4	0	0	0	0	1	0
Spizella_passerina	Land Birds	Emberizidae	0.0122	6030	4	0	0	0	0	1	0
Emberiza_calandra	Land Birds	Emberizidae	0.0488	14700	4	1	1	0	0	1	0
Agelaius_phoeniceus	Land Birds	Icteridae	0.0524	14700	4	1	0	0	0	1	0
Icterus_bullockii	Land Birds	Icteridae	0.0379	12400	4	0	0	0	0	1	0
Regulus_regulus	Land Birds	Regulidae	0.0056	5850	4	0	0	0	0	1	0
Certhia_familiaris	Land Birds	Certhiidae	0.009	7050	4	0	0	0	0	1	0
Salpornis_spilonotus	Land Birds	Certhiidae	0.0148	4180	4	0	0	0	0	1	0
Tichodroma_muraria	Land Birds	Tichodromadidae	0.0176	9410	4	0	0	0	0	1	0
Sitta_europaea	Land Birds	Sittidae	0.0226	16600	4	0	0	0	0	1	0
Polioptila_caerulea	Land Birds	Polioptilidae	0.0058	3890	4	0	0	0	0	1	0
Salpinctes_obsoletus	Land Birds	Troglodytidae	0.0164	10600	4	0	0	0	0	1	0
Campylorhynchus_brunneicapillus	Land Birds	Troglodytidae	0.0389	14700	4	0	0	0	0	1	0

Cistothorus_palustris	Land Birds	Troglodytidae	0.0108	5120	4	1	1	0	1	0	0
Troglodytes_troglodytes	Land Birds	Troglodytidae	0.0098	8810	4	1	1	0	0	1	0
Phainopepla_nitens	Land Birds	Ptilogonatidae	0.0221	7180	4	0	0	0	0	1	0
Bombycilla_garrulus	Land Birds	Bombycillidae	0.0545	17000	4	0	0	0	0	1	0
Cinclus_cinclus	Land Birds	Cinclidae	0.0617	21500	4	0	0	0	0	1	0
Buphagus_erythrorhynchus	Land Birds	Buphagidae	0.0508	8160	4	0	0	0	0	1	0
Lamprotornis_chalybaeus	Land Birds	Sturnidae	0.087	19700	4	0	0	0	0	1	0
Sturnus_vulgaris	Land Birds	Sturnidae	0.0741	40500	4	1	1	0	0	1	0
Onychognathus_morio	Land Birds	Sturnidae	0.133	25600	4	0	0	0	0	1	0
Dumetella_carolinensis	Land Birds	Mimidae	0.0355	13100	4	0	0	0	0	1	0
Mimus_polyglottos	Land Birds	Mimidae	0.0485	15900	4	0	0	0	0	1	0
Toxostoma_redivivum	Land Birds	Mimidae	0.0844	19000	4	0	0	0	0	1	0
Turdus_viscivorus	Land Birds	Turdidae	0.118	29600	4	0	0	0	0	1	0
Turdus_philomelos	Land Birds	Turdidae	0.0678	26000	4	0	0	0	0	1	0
Turdus_migratorius	Land Birds	Turdidae	0.0785	23300	4	0	0	0	0	1	0
Turdus_merula	Land Birds	Turdidae	0.103	26500	4	0	0	0	0	1	0
Muscicapa_striata	Land Birds	Muscicapidae	0.0159	7740	4	0	0	0	0	1	0
Copsychus_saularis	Land Birds	Turdidae	0.036	14100	4	0	0	0	0	1	0
Cercotrichas_coryphaeus	Land Birds	Turdidae	0.0204	6540	4	0	0	0	0	1	0
Cercotrichas_galactotes	Land Birds	Turdidae	0.0203	12900	4	0	0	0	0	1	0
Niltava_sundara	Land Birds	Muscicapidae	0.0211	9410	4	0	0	0	0	1	0
Cossypha_natalensis	Land Birds	Turdidae	0.0341	8810	4	0	0	0	0	1	0
Erithacus_rubecula	Land Birds	Turdidae	0.0177	12300	4	0	0	0	0	1	0
Brachypteryx_leucophrys	Land Birds	Turdidae	0.0158	7880	4	0	0	0	0	1	0
Hodgsonius_phoenicuroides	Land Birds	Turdidae	0.0211	8090	4	0	0	0	0	1	0
Luscinia_brunnea	Land Birds	Turdidae	0.0175	7800	4	0	0	0	0	1	0
Luscinia_megarhynchos	Land Birds	Turdidae	0.0196	10600	4	0	0	0	0	1	0
Tarsiger_cyanurus	Land Birds	Turdidae	0.0135	7000	4	0	0	0	0	1	0
Cinclidium_leucurum	Land Birds	Turdidae	0.027	10900	4	0	0	0	0	1	0
Saxicola_ferrea	Land Birds	Turdidae	0.0149	7870	4	0	0	0	0	1	0
Monticola_saxatilis	Land Birds	Turdidae	0.0503	20000	4	0	0	0	0	1	0
Monticola_cinclorhynchus	Land Birds	Turdidae	0.036	15500	4	0	0	0	0	1	0
Rhyacornis_fuliginosus	Land Birds	Turdidae	0.0204	7600	4	0	0	0	0	1	0
Chaimarrornis_leucocephalus	Land Birds	Turdidae	0.0304	11400	4	0	0	0	0	1	0
Phoenicurus_ochruros	Land Birds	Turdidae	0.0165	9750	4	0	0	0	0	1	0
Phoenicurus_phoenicurus	Land Birds	Turdidae	0.0146	10300	4	0	0	0	0	1	0
