# Supplementary material for: The Evolution of Diapsid Reproductive Strategy with Inferences about Extinct Taxa
Source: PLoS One. 2016 Jul 8;11(7):e0158496. doi: 10.1371/journal.pone.0158496 (PMC4938435; doi:10.1371/journal.pone.0158496)
Supplement: S2 File — (PDF) [file pone.0158496.s002.pdf]

Reproductive Ecology References

Anderson, J. F., A. Hall-Martin, and D. A. Russell. 1985. Long-bone circumference and weight in mammals, birds and dinosaurs. Journal of Zoology London (A)207:53-61.

Antunes, M. T., P. Taquet, and V. Ribeiro. 1998. Upper Jurassic dinosaur and crocodile eggs from Pai Mogo nesting site (Lourinha - Potugal). Memórias da Academia das Cièncias de Lisboa 37:83-99.

Clark, J. M., M. A. Norell, and L. M. Chiappe. 1999. An oviraptorid skeleton from the Late Cretaceous of Ukhaa Tolgod, Mongolia, preserved in an avianlike brooding position over an oviraptorid nest. American Museum Novates 3265:1-36.

Davies, S. J. J. F. 2002. Ratites and Tinamous. S. J. J. F. Davies ed. Oxford University Press, Oxford.

Dong, Z. M., and P. J. Currie. 1996. On the discovery of an oviraptorid skeleton on a nest of eggs at Bayan Mandahu, Inner Mongolia, People's Republic of China. Canadian Journal of Earth Sciences 33:631-636.

Dunning, J. B. Jr. 2008. Avian Body Masses. Page 655 *in* J. B. Dunning, ed. CRC Press, Boca Raton.

Erickson, G. M., K. C. Rogers, D. J. Varricchio, M. A. Norell, and X. Xu. 2007. Growth patterns in brooding dinosaurs reveals the timing if sexual maturity in non-avian dinosaurs and genesis of the avian condition. Biology Letters 3:558-561.

Ferguson, M. W. J. 1985. Reproductive biology and embryology of the crocodilians. Page 451–460 *in* C. Gans, F. Billet, and P. F. A. Maderson ed. Biology of the Reptilia, John Wiley and Sons, New York.

Grellet-Tinner G., Chiappe, L. M., Norell, M. and Bottjer, D. 2006. Dinosaur eggs and nesting ecology: A paleobiological investigation. Palaeogeography, Palaeoclimatology, Palaeoecology 232:294-321.

Hackett, S. J., R. T. Kimball, S. Reddy, R. C. K. Bowie, E. L. Braun, M. J. Braun, J. L. Chojnowski, W. A. Cox, K. L. Han, J. Harshman, C. J. Huddleston, B. D. Marks, K. J. Miglia, W. S. Moore, F. H. Sheldon, D. W. Steadman, C. C. Witt, T. Yuri. 2008. A phylogenomic study of birds reveals their evolutionary history. Science, 320:1763-1767.

Heinroth, O. 1922. Die Beziehungen zwischen Vogelgewicht, Eigewicht, Gelegegewicht und Brutdauer. Journal of Ornithology 70:172-285.

Henderson, D. M. 1999. Estimating the masses and centers of mass of extinct animals by 3-D mathematical slicing. Paleobiology 25:88–106.

Hirsch, K.F. and B. Quinn. 1990. Eggs and eggshell fragments from the Upper Cretaceous Two Medicine Formation of Montana. Journal of Vertebrate Paleontology 10:491-511.

Horner, J. R. 1982. Evidence of colonial nesting and 'site fidelity' among ornithischian dinosaurs. Nature 297:675-676.

Horner, J. R. 1984. The nesting behavior of dinosaurs. Scientific American 250(4):130-137.

Horner, J. R. 1999. Egg clutches and embryos of two hadrosaurian dinosaurians. Journal of Vertebrate Paleontology 19:607-611.

Hoyt, D. F. 1979. Practical methods of estimating volume and fresh weight of bird eggs. Auk 96:73-77.

Iverson, J. B. and M. A. Ewert. 1991. Physical characteristics of reptilian eggs and a comparison with avian eggs. Pages 87-100 *in* D. C. Deeming and M. W. J. Ferguson ed. Egg Incubation, Its

Effects on Embryonic Development in Birds and Reptiles. Cambridge University Press, Cambridge.

Jackson, F. D., D. J. Varricchio, R. Jackson, B. Vila, and L .Chiappe. 2008. Water vapor conductance of a titanosaur egg (Megaloolithus patagonicus) from Argentina: comparison with a *Megaloolithus siruguei* egg from Spain. Paleobiology 34:229-246.

Jarvis, E. D. et al. 2014. Whole-genome analysis resolve early branches in the tree of life of modern birds. Science 346:1320-1331.

Jones, D. N., R. W. R. J. Dekker, and C. S. Roselaar. 1995. The Megapodes, Megapodiidae. D. N. Jones, R. W. R. J. Dekker and C. S. Roselaar, ed. Oxford University Press, Oxford.

Kitching, J. W. 1979. Preliminary report on a clutch of six dinosaurian eggs from the Upper Triassic Elliot Formation, Northern Orange Free State. Palaeontologia Africana 22:41-45.

Mateus, I. H., M. T. Antunes, O. Mateus, P. Tacquet, V. Ribeiro, and G. Manuppella. 1997. Couvee, oeufs et embryons d'un dinosaure theropode du Jurassique superieur de Lourinha. Comptes Rendus de l'Academie des Sciences de Paris 325(A):71-78.

Mikhailov, K. E., K. Sabath, and S. Kurzanov. 1994. Eggs and nests from the Cretaceous of Mongolia. Pages 88-115 *in* K. Carpenter, K.F. Hirsch, and J.R. Horner ed. Dinosaur Eggs and Babies. Cambridge University Press, New York.

Norell, M. A., J. M. Clark, L. M. Chiappe, and D. Dashzeveg. 1995. A nesting dinosaur. Nature 378:774-776.

Oring, Lewis W., E. M. Gray, and J. M. Michael Reed. 1997. Spotted Sandpiper (*Actitis macularius*), The Birds of North America Online. A. Poole, Ed. Ithaca: Cornell Lab of Ornithology; the Birds of North America Online.

Paganelli, C. V., A. Olszowka, and A. Ar. 1974. The avian egg: surface area, volume, and density. Condor 76:319-325.

Paul, G. S. 1988. Predatory Dinosaurs of the World: A Complete Illustrated Guide. G. S. Paul ed. Simon and Schuster, New York.

Peczkis, J. 1994. Implications of body-mass estimates for dinosaurs. Journal of Vertebrate Paleontology 14:520-533.

Reisz, R. R., D. Scott, H.-D. Sues, D. C. Evans, and M. A. Raath. 2012. Embryos of an Early Jurassic prosauropod dinosaur and their evolutionary significance. Science 309:761-764.

Russell, D. A. 1969. A new specimen of Stenonychosaurus from the Oldman Formation (Cretaceous) of Alberta. Canadian Journal of Earth Sciences 6:595-612.

Russell, D. A., and R. Séguin. 1982. Reconstruction of the small Cretaceous theropod *Stenonychosaurus inequalis* and a hypothetical dinosauroid. Syllogeus 37:1-43.

Sabath K. 1991. Upper Cretaceous amniotic eggs from the Gobi Desert. Palaeontologica Polonica. 36:151-192.

Sander, P. M., C. Peitz, F. Jackson, and L. Chiappe. 2008. Upper Cretaceous titanosaur nesting sites and their implications for sauropod reproductive biology. Palaentographica (A) 284:69-107.

Seddon, N., J. A. Tobias, and S. H. M. Butchart. 2003. Group living, breeding behaviour and territoriality in the Subdesert Mesite *Monias benschi*. Ibis 145:277–294.

Shine, R. 1988. Parental care in reptiles. Pages 276-329 in C. Gans and R.B. Huey ed. Biology of the Reptilia, Volume 16, Ecology B., Branta Books, Ann Arbor, Michigan.

Thorbjarnarson, J. B. 1996. Reproductive characteristics of the order Crocodylia. Herpetologica 52:8-24.

Trutnau, L. and Sommerlad, R. 2006. Crocodilians: their natural history and captive husbandry. L. Trunau and R. Sommerlad ed. Chimaira, Frankfurt.

Varricchio, D. J., Jackson, F. D., Borkowski, J. and Horner, J. R. 1997. Nest and egg clutches of the dinosaur *Troodon formosus* and the evolution of avian reproductive traits. Nature 385:247-250.

Varricchio, D. J., Jackson, F.D., Jackson, R.A., and Zelenitsky, D.K. 2013. Porosity and water vapor conductance of two *Troodon formosus* eggs: an assessment of incubation strategy in a maniraptoran dinosaur. Paleobiology 39(2):278–296.

Walters, M. 1994. *Birds' Eggs*. M. Walters Ed. Dorling Kindersley, London.

Yang, R., X. Wu, P. Yan, X. Su, and B. Yang. 2010. Complete mitochondrial genome of *Otis tarda* (Gruiformes: Otididae) and phylogeny of Gruiformes inferred from mitochondrial DNA sequences. Molecular Biology Reports 37:3057-3066.
