# Supplementary material for: The Evolution of Diapsid Reproductive Strategy with Inferences about Extinct Taxa
Source: PLoS One. 2016 Jul 8;11(7):e0158496. doi: 10.1371/journal.pone.0158496 (PMC4938435; doi:10.1371/journal.pone.0158496)
Supplement: S3 File — (PDF) [file pone.0158496.s003.pdf]

Supplementary File 2 - Phylogeny References

Alström, P., P. G. P. Ericson. 2006. Phylogeny and classification of the avian superfamily Sylvioidea. Molecular Phylogenetics and Evolution 38:381-397.

Barker, F. K., G. F. Barroclough, and J. G. Groth. 2002. A phylogenetic hypothesis for passerine birds: Taxonomic and biogeographic implications of an analysis of nuclear DNA sequence data. Proceedings of the Royal Society of London B 269:295-308.

Bertelli, S., and A. L. Porzecanski. 2004. Tinamou (Tinamidae) systematics: A preliminary combined analysis of morphology and molecules. Ornitologia Neotropical 15(suppl.):1-7.

Belbic, A. J., I. Seibolc, W. Bednarek, P. Gaucher, D. Ristow, W. Scharlau, D. Schmidl, and M. Wink. 1994. Phylogenetic relationships among Falcon species (genus *Falco*) according to DNA sequence variation of the cytochrome b gene. *In* B. Meyburg, and R. D. Chancellor ed. Raptor Conservation Today 593-599.

Bridge, E. S., A. W. Jones, and A. J. Baker. 2005. A phylogenetic framework for the terns (Sternini) inferred from mtDNA sequences: Implications for taxonomy and plumage evolution. Molecular Phylogenetics and Evolution 35:459-469.

Chesser, R. T. 2004. Molecular systematics of New World suboscine birds. Molecular Phylogenetics and Evolution 35:11-24.

Crowe, T. M., P. Bloomer, E. Randi, V. Lucchini, R. Kimbball, E. Braun, and J. G. Groth. 2006. Supra-generic cladistics of landfowl (Order Galliformes). Acta Zoologica Sinica 52(suppl.):358-361.

Dowling, H. G., C. A. Hass, B. S. Hedges, and R. Highton. 1996. Snake relationships revealed by slow-evolving proteins: A preliminary survey. Journal of the Zoological Society of London 240:1-28.

Driskell, A., L. Christidis, B. J. Gill, W. E. Boles, F. K. Barker, and N. W. Longmore. 2007. A new endemic family of New Zealand passerine birds: Adding heat to a biodiversity hotspot. Australian Journal of Zoology 55:73-78.

Fain, M. G., and P. Houde. 2007. Multilocus perspectives on the monophyly and phylogeny of the order Charidriiformes (Aves). BMC Evolutionary Biology, 7(35):15.

Fjeldså, J., M. Irestedt, P. G. P. Ericson, and D. Zuccon. 2010. The Cinnamon Ibon *Hypocryptadius cinnamomeus* is a forest canopy sparrow. Ibis 152:747-760.

Gelang, M., A. Cibois, E. Pasquet, U. Olsson, P. Alström, and P. G. P. Ericson. 2009. Phylogeny of babblers (Aves, Passeriformes): Major lineages, family limits and classification: Zoologica Scripta 38:225-236.

Gonzalez, J., H. Duttman, and M. Wink. 2009. Phylogenetic relationships based on two mitochondrial genes and hybridization patterns in Anatidae. Journal of Zoology 279:310-318.

Griffiths, C. S., G. F. Barrowclough, J. G. Groth, and L. Mertz. 2004. Phylogeny of the Falconidae (Aves): A comparison of the efficacy of morphological, mitochondrial, and nuclear data. Molecular Phylogenetics and Evolution 32:101-109.

Griffiths, C. S., G. F. Barrowclough, J. G. Groth, and L. A. Merts. 2007. Phylogeny, diversity, and classification of the Accipitridae based on DNA sequences and the RAG-1 exon. Journal of Avian Biology 38:587-602.

Hackett, S. J., R. T. Kimball, S. Reddy, R. C. K. Bowie, E. L. Braun, M. J. Braun, J. L. Chojnowski, W. A. Cox, K. L. Han, J. Harshman, C. J. Huddleston, B. D. Marks, K. J. Miglia, W. S. Moore, F. H. Sheldon, D. W. Steadman, C. C. Witt, T. Yuri. 2008. A phylogenomic study of birds reveals their evolutionary history. Science, 320:1763-1767.

Hall, K. S. S., and B. S. Tullberg. 2004. Phylogenetic analysis of the diversity of moult strategies in Sylviidae in relation to migration. Evolutionary Ecology 18:85-105.

Han, K.-L., M. B. Robbins, and M. J. Braum. 2010. A multi-gene estimate of phylogeny in the nightjars and nighthawks (Caprimigulidae). Molecular Phylogenetics and Evolution 55(2):443-453.

Irestedt, M., U. S. Johansson, T. J. Parsons, and P. G. P. Ericson. 2001. Phylogeny of major lineages of suboscine (Passeriformes) analyzed by nuclear DNA sequence data. Journal of Avian Biology 32:15-25.

Jarvis, E. D. et al. 2014. Whole-genome analysis resolve early branches in the tree of life of modern birds. Science 346:1320-1331.

Johansson, U. S., J. Fjeldså, and R. C. K. Bowie. 2008. Phylogenetic relationships within Passerida (Aves: Passeriformes): A review and a new molecular phylogeny based on three nuclear intron markers. Molecular Phylogenetics and Evolution 48:858-876.

Johnson, K. P., and D. H. Clayton. 2000: Nuclear and mitochondrial genes contain similar phylogenetic signal for pigeons and doves (Aves: Columbiformes). Molecular Phylogenetics and Evolution 14(1):141-151.

Jønsson, K. A., and J. Fjeldså. 2006. A phylogenetic supertree of oscine passerine birds (Aves: Passeri). Zoologica Scripta 35(2):149-186.

Kelly, C. M. R., N. P. Barker, M. H. Villet, and D. G. Broadley. 2009. Phylogeny, biogeography and classification of the snake superfamily Elapoidea: A rapid radiation in the late Eocene. Cladistics 25:33-63.

Kennedy, M., and R. D. M. Page. 2002. Seabird supertrees: Combining partial estimates of Procellariiform phylogeny. The Auk 119(1):88-108.

Kennedy, M., and H. G. Spencer. 2004. Phylogenies of the Frigatebirds (Fregatidae) and Tropicbirds (Phaethonidae), two divergent groups of the traditional order Pelecaniformes, inferred from mitochondrial DNA sequences. Molecular Phylogenetics and Evolution 31:31-38.

Klicka, J., K. Burns, and G. M. Spellman. 2007. Defining a monophyletic Cardinalini: A molecular perspective. Molecular Phylogenetics and Evolution 45:1014-1032.

Krajewski, C., and J. W. Fetzner, Jr. 1994. Phylogeny of cranes (Gruiformes: Gruidae) based on Cytochrome-B DNA sequences. The Auk 111(2):351-365.

Ksepka, D. T., S. Bertelli, and N. P. Giannini. 2006. The phylogeny of the living and fossil Sphenisciformes (penguins). Cladistics 22:412-441.

Larsen, C., Speed, M., N. Harvey, and H. A. Noyes. 2007. A molecular phylogeny of the nightjars (Aves: Caprimigulidae) suggests extensive conservation of primitive morphological traits across multiple lineages. Molecular Phylogenetics and Evolution 42:789-796.

Livezey, B. C. 2010. Phylogenetics of modern shorebirds (Charidriiformes) based on phenotypic evidence: Analysis and discussion. Zoological Journal of the Linnean Society 160:567-618.

Malone, C. L., T. Wheeler, J. F. Taylor, and S. K. Davis. 2000. Phylogeography of the Caribbean Rock Iguana (Cyclura): Implications for conservation and insights on the biogeographic history of the West Indies. Molecular Phylogenetics and Evolution 17(2):269-279.

Mayr, G., 2002. Osteological evidence for paraphyly of the avian order Caprimiguliformes (nightjars and allies). Journal of Ornithology 143:82-97.

Melville, J., and J. M. Hale. 2009. Length variation in the N-terminal domain of the recombination-activating gene 1 (RAG1) across squamates. Molecular Phylogenetics and Evolution 52:898-903.

Moyle, R., G., R. T. Chesser, R. O. Prum, P. Schikler, and J. Cracraft. 2006. Phylogeny and evolution history of Old World Suboscine birds (Aves: Eurylaimides). American Museum Novitates 3544:22

Moyle, R. G., J. Cracraft, M. Lakim, J. Nais, and F. H. Sheldon. 2006. Reconsideration of the phylogenetic relationships of the enigmatic Bornean Bristlehead (*Pityriasis gymnocephala*). Molecular Phylogenetics and Evolution 39:893-898.

Moyle, R. G., R. T. Chesser, R. T. Brumfield, J. G. Tello, D. J. Marchese, and J. Cracraft. 2009. Phylogeny and phylogenetic classification of the antbirds, ovenbirds, woodcreepers, and allies (Aves: Passeriformes: infraorder Furnariides). Cladistics 25:386-405.

Naro-Maciel, E., M. Le, N. N. Fitz Simmons, and G. Amato. 2008. Evolutionary relationships of marine turtles: A molecular phylogeny based on nuclear and mitochondrial genes. Molecular Phylogenetics and Evolution 49:659-662.

Nylander, J. A. A., U. Olsson, P. Alstrom, and I. Sanmartin. 2008. Accounting for phylogenetic uncertainty in biogeography: A Baysian approach to dispersal-vicariance analysis of the thrushes (Aves: *Turdus*). Systematic Biology 57(2):257-268.

Oakes, E. J.1992. Lekking and the evolution of sexual dimorphism in birds: Comparative approaches. The American Naturalist 140(4):665-684.

Ohlson, J., J. Fjeldså, and P. G. P. Ericson. 2008. Tyrant flyscatchers coming out in the open: phylogeny and ecological radiation of Tyrannidae (Aves, Passeriformes). Zoologica Scripta 37(3):315-335.

Pasquet, E., J. M. Pons, J. Fuchs, C. Cruaud, and V. Bretagnolle. 2007. Evolutionary history and biogeography of the drongos (Dicruridae), a tropical Old World clade of corvid passerines. Molecular Phylogenetics and Evolution 45:158-167.

Pereira, S. L., A. L. Baker. 2008. DNA evidence for a Paleocene origin of the Alcidae (Aves: Charidriiformes) in the Pacific and multiple dispersals across northern oceans. Molecular Phylogenetics and Evolution 46:430-445.

Pitra, C., D. Leickfeldt, S. Frahnert, and J. Fickel. 2002. Phylogenetic relationships and ancestral areas of the bustards (Gruiformes: Otididae), inferred from mitochondrial DNA and nuclear intron sequences. Molecular Phylogenetics and Evolution 23(1):63-74.

Poe, S. 1996. Data set incongruence and the phylogeny of crocodilians: Systematic Biology 45(4):393-414.

Pons, J.-M., A. Hassanin, and P. A. Crochet. 2005. Phylogenetic relationships within the Laridae (Charidriiformes: Aves) inferred from mitochondrial markers. Molecular Phylogenetics and Evolution 35:686-699.

Pyron, R. A., F. T. Burbrink, G. R. Colli, A. N. Montes de Oca, L. J. Vitt, C. A. Kuczynski, and J. J. Weins. 2010. The phylogeny of advanced snakes (Colubroidea), with discovery of a new subfamily and comparison of support methods for likelihood trees. Molecular Phylogenetics and Evolution 58(2):329-342.

Rawlings, L. H., D. L. Rabosky, S. C. Donnellan, and M. N. Hutchinson. 2008. Python phylogenetics: Inference from morphology and mitochondrial DNA. Biological Journal of the Linnean Society 93:603-619.

Sangster, G., J. M. Collinson, A. G. Knox, D. T. Parkin, and L. Svensson. 2010. Taxonomic recommendations for British birds: Sixth report. Ibis 152:180-186.

Sheldon, F. H., and B. Slikas. 1997. Advances in Ciconiiform systematics 1976-1996. Colonial Waterbirds 20(1):106-114.

Sheldon, F. H.1987. Phylogeny of Herons estimated form DNA-DNA hybridization data. The Auk 104(1):97-108.

Sibley, C. G., and J. E. Ahlquist. 1990. Phylogeny and classification of birds. C.G. Sibley and J. E. Ahlquist ed. New Haven, Yale University Press.

Slikas, B., S. L. Olson, and R. C. Fleischer. 2002. Rapid, independent evolution of flightlessness in four species of Pacific Island rails (Rallidae): an analysis based on mitochondrial sequence data. Journal of Avian Biology 33:5-14.

Sorenson, M. D., and R. B. Payne. 2001. A single ancient origin of brood parasitism in African finches: Implication so host-parasite coevolution. Evolution 55(12):2550-2567.

Thomas, G. H., M. A. Wills, and T. Székely. 2004. A supertree approach to shorebird phylogeny: BMC Evolutionary Biology 4(28):18.

Thomson, R. C. and H. B. Shaffer. 2010. Sparse supermatrices for phylogenetic inference: Taxonomy, alignment, rogue taxa and the phylogeny of living turtles. Systematic Biology 59(1):42-58.

Treplin, S., R. Seigert, C. Bleidom, H. S. Thompson, R. Fotso, and R. Tiedemann. 2008. Molecular phylogeny of songbirds (Aves: Passeriformes) and the relative utility of common nuclear marker loci. Cladistics 24:328-349.

Trewick, S. A.1997. Flightlessness and phylogeny amongst endemic rails (Aves: Rallidae) of the New Zealand region. Philosophical Transactions of the Royal Society of London B. 352:429-446.

Voelker, G., and S. V. Edwards. 1998. Can weighting improve bushy trees? Models of Cytochrome b evolution and the molecular systematics of pipits and wagtails (Aves: Motacillidae). Systematic Biology 47(4):589-603.

Walther, B. A., and D. H. Clayton. 2005. Elaborate ornaments are costly to maintain: evidence for high maintenance handicaps. Behavioral Ecology 16(1):89-95.

Wink, M. 1995. Phylogeny of Old and New World vultures (Aves: Accipitridae and Cathartidae) inferred from nucleotide sequences of the mitochondrial Cytochrome b gene. Verlag der Zeitschrift für Naturforschung 50:868-882.

Wink, M. and I. Seibold. 1996: Molecular phylogeny of Mediterranean raptors (families Accipitridae and Falconidae). *Biologla y Conservación de las Rapaces Mediterráneas* 334-344.

Yang, R., X. Wu, P. Yan, X. Su, and B. Yang. 2010. Complete mitochondrial genome of *Otis tarda* (Gruiformes: Otididae) and phylogeny of Gruiformes inferred from mitochondrial DNA sequences. Molecular Biology Reports 37:3057-3066.

Yom-Tov, Y., and E. Geffen. 2005. On the origin of brood parasitism in altricial birds. Behavioral Ecology 17(2):196-206.

Zuccon, D., and P. G. P. Ericson. 2010. A multi-gene phylogeny disentangels the chat-flycatcher complex (Aves: Muscicapidae). Zoologica Scripta 39(3):213-224.
