# Supplementary material for: The Evolution of Diapsid Reproductive Strategy with Inferences about Extinct Taxa
Source: PLoS One. 2016 Jul 8;11(7):e0158496. doi: 10.1371/journal.pone.0158496 (PMC4938435; doi:10.1371/journal.pone.0158496)
Supplement: S5 File — (PDF) [file pone.0158496.s005.pdf]

DERIVED THEROPODS

	Body Mass (kg)	Clutch Volume (mm3)	Log10 BM	Log10 CV	Precociality	No Care Predicted Clutch Volume	Sum of Squares	Maternal Care Predicted Clutch Volume	Sum of Squares	Paternal Care Predicted Clutch Volume	Sum of Squares	Biparental Care Predicted Clutch Volume	Sum of Squares
Citipati osmolskae	79.23	8767000	1.898889656	6.942851007	1	6.362626357	0.336660644	6.581973987	0.130232223	6.760343147	0.033309119	6.632635937	0.096233389
Machairasaurus philoceratops	39	7140000	1.591064607	6.853698212	1	6.151480944	0.493109092	6.370828574	0.233163088	6.549197734	0.092720541	6.421490524	0.186803486
Troodon formosus	51.4	7114000	1.710963119	6.852113861	1	6.233722532	0.382407836	6.453070162	0.159235874	6.631439322	0.048697252	6.503732112	0.121369843
Byronosaurus jaffei	22.5	3240000	1.352182518	6.51054501	1	5.987625345	0.273444976	6.206972975	0.09215598	6.385342135	0.01567576	6.257634925	0.063963511

	Total Sum of Squares	AIC	Delta AIC	Relative likelihood	Wi	Evidence ratio
No Care	1.485622547	3.891664858	6.163344094	0.045882475	0.031055573	21.7948139
Maternal Care	0.614787165	1.244725699	3.516404935	0.172354398	0.116658148	5.801998735
Paternal Care	0.190402672	-2.271679236	0	1	0.67685043	
Biparental Care	0.468370229	0.428674509	2.700353745	0.259194412	0.175435849	3.858107864
				1.477431285		

HADROSAURS

	Body Mass (kg)	Clutch Volume (mm3)	Log10 BM	Log10 CV	Precociality	No Care Predicted Clutch Volume	Sum of Squares	Maternal Care Predicted Clutch Volume	Sum of Squares	Paternal Care Predicted Clutch Volume	Sum of Squares	Biparental Care Predicted Clutch Volume	Sum of Squares
Lambeosaurine	2042	92180000	3.310055738	7.964636704	3	7.138738843	0.682107277	7.358086473	0.367903183	7.536455633	0.18333903	7.408748423	0.309011781
Maiasaura peeblesorum	2408	14400000	3.381656483	7.158362492	3	7.187851704	0.000869614	7.407199334	0.061919774	7.585568494	0.182504968	7.457861284	0.089699526

	Total Sum of Squares	AIC	Delta AIC	Relative likelihood	Wi	Evidence ratio
No Care	0.68297689	1.560280368	1.872762051	0.392044069	0.128276997	2.550733655
Maternal Care	0.429822957	0.171017482	0.483499166	0.785252794	0.256935071	1.273475253
Paternal Care	0.365843998	-0.312481683	0	1	0.327200455	
Biparental Care	0.398711307	-0.05438986	0.258091824	0.878933611	0.287587477	1.137742359
				3.056230474		

SAUROPODOMORPHS

	Body Mass (kg)	Clutch Volume (mm3)	Log10 BM	Log10 CV	Precociality	No Care Predicted Clutch Volume	Sum of Squares	Maternal Care Predicted Clutch Volume	Sum of Squares	Paternal Care Predicted Clutch Volume	Sum of Squares	Biparental Care Predicted Clutch Volume	Sum of Squares
Massospondylus carinatus	107	678000	2.029383778	5.831229694	1	6.452135757	0.385524339	6.671483387	0.706026269	6.849852547	1.037592517	6.722145337	0.793730683
Megaloolithus patagonicus	5000	38500000	3.698970004	7.58546073	1	7.597349494	0.000141343	7.816697124	0.05347027	7.995066284	0.167776711	7.867359074	0.079466677
Megaloolithus sirguei	10000	41500000	4	7.618048097	1	7.803834	0.034516402	8.02318163	0.16413318	8.20155079	0.340475393	8.07384358	0.207749523

	Total Sum of Squares	AIC	Delta AIC	Relative likelihood	Wi	Evidence ratio
No Care	0.420182084	0.102961748	0	1	0.591399534	
Maternal Care	0.923629719	2.46583106	2.362869312	0.306838215	0.181463978	3.259046461
Paternal Care	1.545844621	4.010874457	3.907912709	0.141712297	0.083808586	7.056550642
Biparental Care	1.080946883	2.937675335	2.834713587	0.242353762	0.143327902	4.126199615
				1.690904274		
