# Supplementary figures and images for: The Evolution of Diapsid Reproductive Strategy with Inferences about Extinct Taxa
Source: PLoS One. 2016 Jul 8;11(7):e0158496. doi: 10.1371/journal.pone.0158496 (PMC4938435; doi:10.1371/journal.pone.0158496)

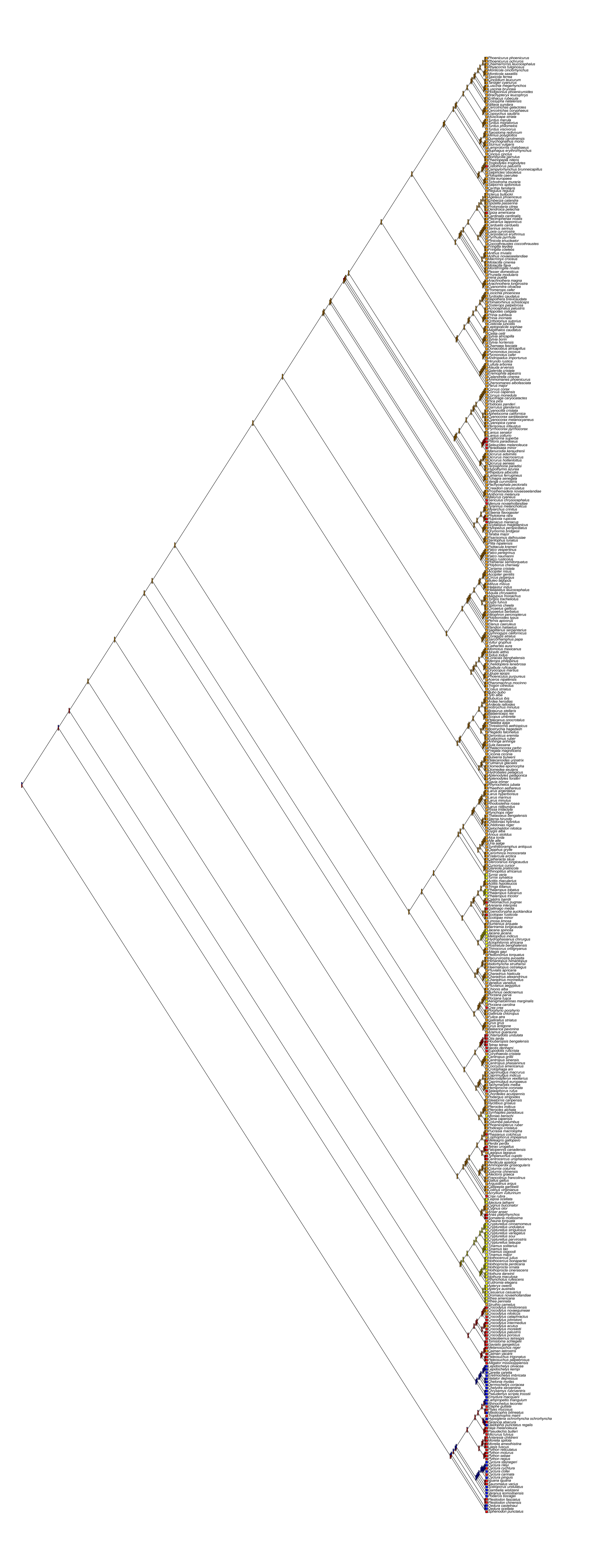

Supplement: S6 File — The proportions of colors in each the bars at each node of the phylogeny represents the likelihood of each care strategy at that node. Blue = no care, red = maternal care, orange = biparental care, yellow = paternal care. (PDF) [file pone.0158496.s006.pdf]

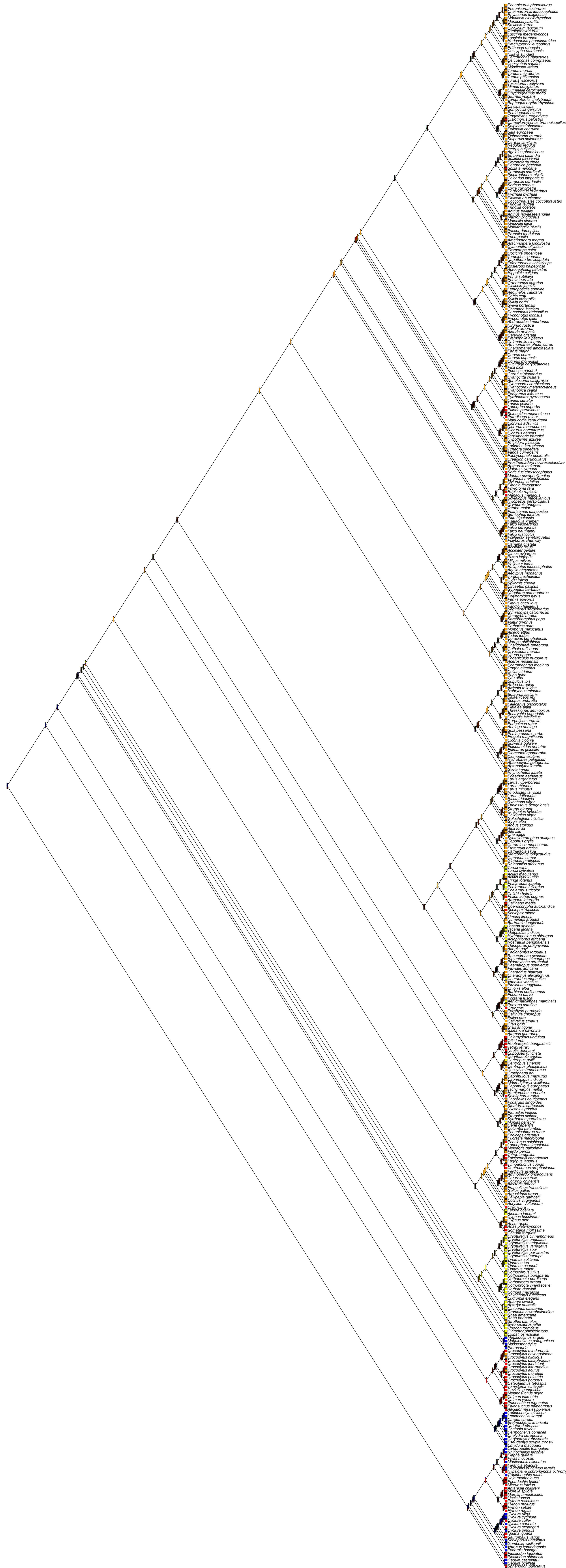

Supplement: S7 File — The proportions of colors in each the bars at each node of the phylogeny represents the likelihood of each care strategy at that node. Blue = no care, red = maternal care, orange = biparental care, yellow = paternal care. (PDF) [file pone.0158496.s007.pdf]

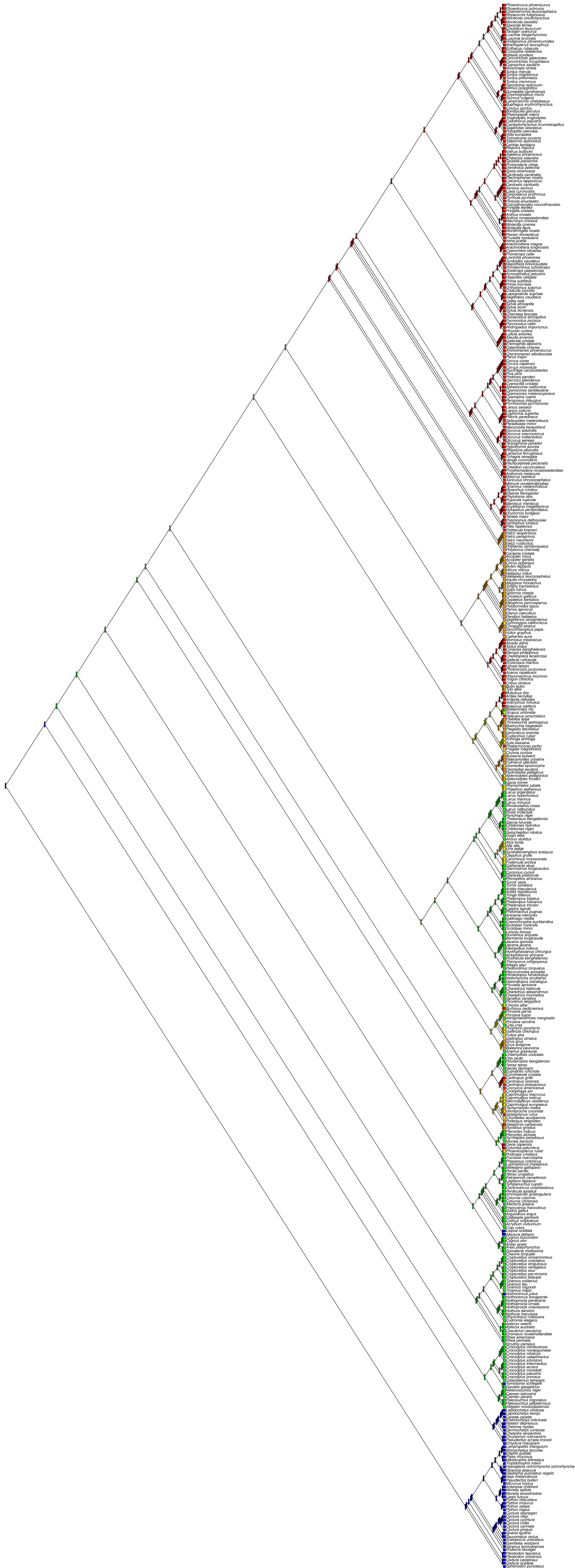

Supplement: S8 File — The proportions of colors in each the bars at each node of the phylogeny represents the likelihood of each care strategy at that node. Blue = no care, red = maternal care, orange = biparental care, yellow = paternal care. Light blue = superprecocial, green = precocial, yellow = semiprecocial, orange = subaltricial, red = altricial. (PDF) [file pone.0158496.s008.pdf]

A

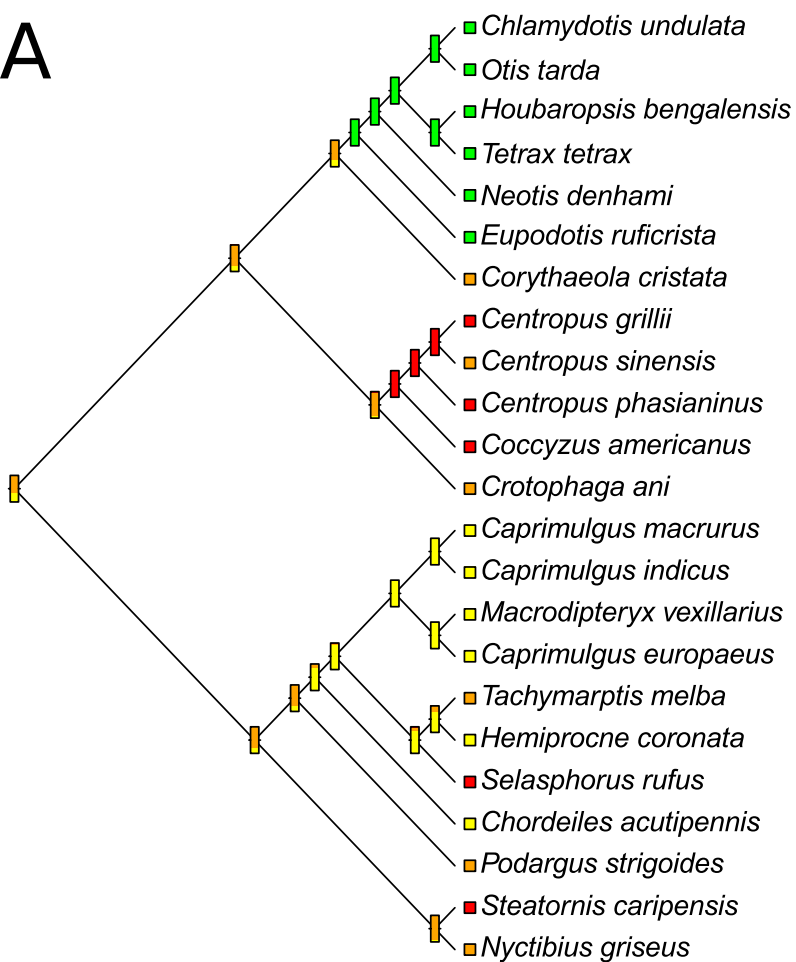

B

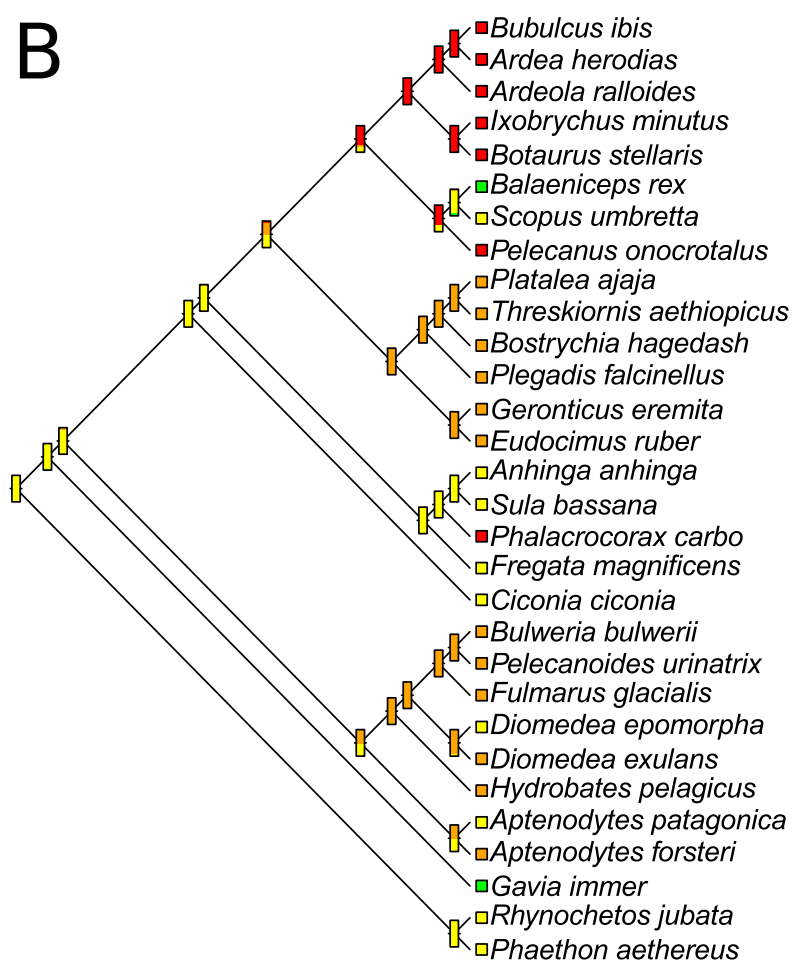

Supplement: S9 File — A–Caprimulgimorpha + Otidimorphae, B–Aequiornithia + Phaethontimorphae. Green = precocial, yellow = semiprecocial, orange = subaltricial, red = altricial. The proportion of the colored bar at each node represents the likelihood of each precociality state at that node. (PDF) [file pone.0158496.s009.pdf]

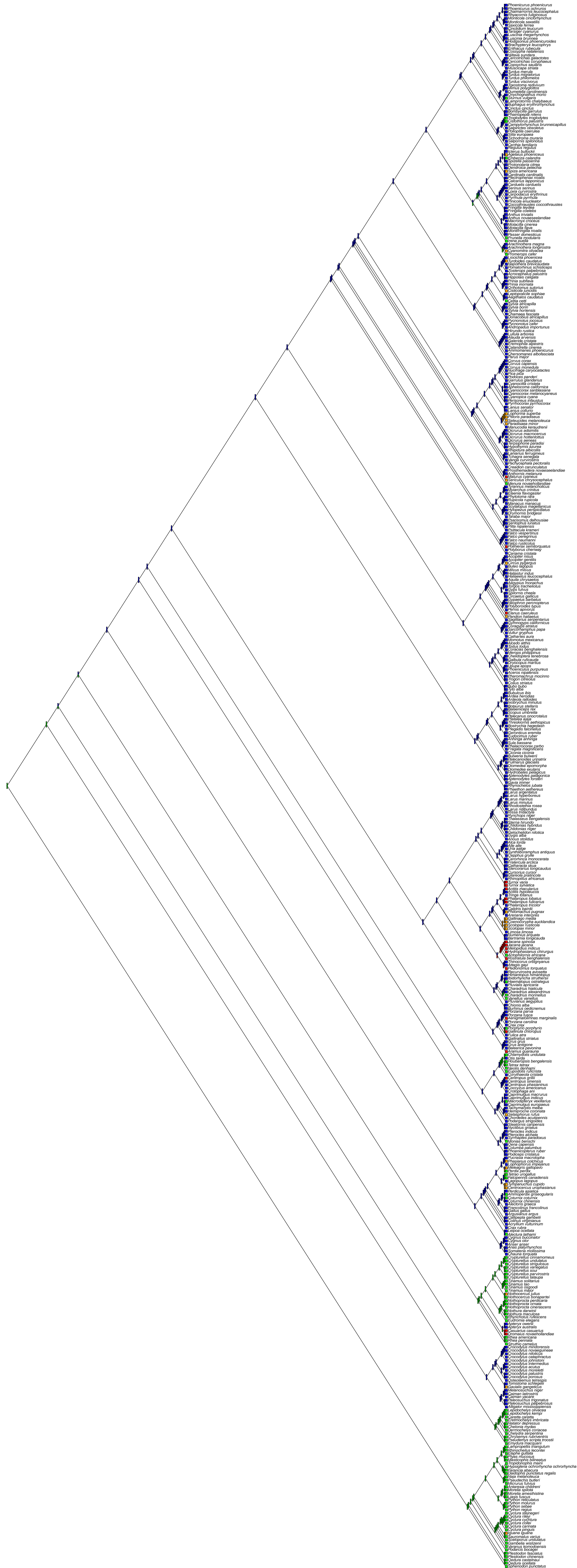

Supplement: S10 File — The proportions of colors in each the bars at each node of the phylogeny represents the likelihood of each care strategy at that node. Green = both sexes polygamous, blue = socially monogamous. (PDF) [file pone.0158496.s010.pdf]
